# Targeting HER3 or MEK overcomes acquired Trastuzumab resistance in HER2-positive gastric cancer-derived xenograft

**DOI:** 10.1038/s41420-022-01259-z

**Published:** 2022-12-03

**Authors:** Mengqi Zhang, Beifang Li, Haiyan Liao, Zuhua Chen, Wenwen Huang, Jing Yang, Sai Ge, Zhongwu Li, Lin Shen, Cheng Zhang, Jing Gao

**Affiliations:** 1 Key laboratory of Carcinogenesis and Translational Research (Ministry of Education/Beijing), Department of Gastrointestinal Oncology, Peking University Cancer Hospital and Institute, Beijing, 100142 China; 2 Department of Oncology, The First Affiliated Hospital, Sun Yat-sen University, Guangzhou, 510080 China; 3 Institute of Precision Medicine, The First Affiliated Hospital, Sun Yat-sen University, Guangzhou, 510080 China; 4 Department of Oncology, Shenzhen Key Laboratory of Gastrointestinal Cancer Translational Research, Cancer Institute, Peking University Shenzhen Hospital, Shenzhen-Peking University-Hong Kong University of Science and Technology Medical Center, Shenzhen, 518036 China; 5 Key laboratory of Carcinogenesis and Translational Research (Ministry of Education/Beijing), Department of Pathology, Peking University Cancer Hospital and Institute, Beijing, 100142 China; 6 National Cancer Center/National Clinical Research Center for Cancer/Cancer Hospital & Shenzhen Hospital, Chinese Academy of Medical Sciences and Peking Union Medical College, Shenzhen, 518116 China

**Keywords:** Gastric cancer, Translational research

## Abstract

Acquired Trastuzumab resistance is a complicated and disastrous event for HER2-positive gastric cancer (GC). In this study, we successfully established a GC PDX model with Trastuzumab sensitivity (176P) and induced a homologous model with acquired Trastuzumab resistance (176R), then comprehensively delineated the landscape of Trastuzumab resistance mechanisms using single-cell transcriptome sequencing, as well as protein profiling and genomic variation analysis. According to multi-omics study, different gene expression profiles, rather than genetic changes, contributed to acquired Trastuzumab resistance. The mechanisms underlying acquired Trastuzumab resistance present great complexity as multiple molecules and pathways were involved, including ERBB family, MAPK, PI3K/AKT, JAK/STAT, and cell cycle pathways. Through phenotypical and molecular validation, we found that Trastuzumab combined with HER3-targeted antibody or MEK inhibitor demonstrated excellent antitumor activity and good tolerance, which may serve as promising strategies for overcoming acquired Trastuzumab resistance.

## Introduction

As the fifth most prevalentcancer worldwide, gastric cancer (GC) is highly malignant and harbors extremely poor prognosis [1–3]. The treatment strategy for GC is limited due to lack of therapeutic targets, HER2 (ERBB2) is one of the few options. Despite the clinical benefit of Trastuzumab combined with chemotherapy in improving the overall survival of HER2-positive advanced GC (AGC), the disease progressed in most patients within one year because of acquired Trastuzumab resistance [4, 5]. Several second-line treatment options after Trastuzumab resistance such as T-DM1 or Lapatinib responded in breast cancer but failed in GC [6–8]. Therefore, it was urgent to explore mechanisms of acquired Trastuzumab resistance and to develop overcoming strategies for improving survival of advanced GC patients.

According to previous reports, the reactivation of PI3K/AKT, MAPK and other downstream pathways primarily inhibited by HER2-blocking was a key mechanism responsible for acquired Trastuzumab resistance [9, 10]. Also, MET amplification and overexpression of EphA2, IGF1R, CyclinD1 might lead to Trastuzumab resistance in breast cancer [11–14]. As recently reported, liquid biopsy-identified mutation of PIK3CA/HER2/NF1, increased ERBB2 copy number as well as upregulation of ERBB-family ligands, can induce Trastuzumab resistance in gastric cancer [15, 16]. Even so, the definite mechanism of acquired Trastuzumab resistance remains unclear and reliable strategy for overcoming resistance is as yet lacking in gastric cancer, majorly due to the deficiency of optimal preclinical anthropomorphic models and multi-dimensional systematic study followed by validation of overcoming resistance strategies.

In recent years, ex vivo models represented by PDX (patient-derived xenograft) and organoid become a new favorite in preclinical research due to their high fidelity with patient characteristics [17, 18]. In China, more than 70% GC patients are diagnosed as AGC and lose the opportunity of radical surgery, thus endoscopic biopsy is the primary method to obtain tumor tissues [19, 20]. In our previous studies, we successfully established a comprehensive library of PDX models covering a variety of types using biopsy tissues from AGC patients [18]. Based on certain Trastuzumab sensitive PDX models, we mirrored patient-sourced medication and successfully induced one Trastuzumab acquired resistant PDX model through intermittent stimulation. According to current reports, this was currently the first available pair of Trastuzumab sensitive and resistant PDX models that came from a same patient sample.

It was well known that GC was featured by high tumor heterogeneity, and subclonal selection might be the main reason for drug resistance [21]. Many studies had shown that omics method was an ideal approach to reveal intratumor-heterogeneity and elucidate drug resistant mechanism [22–24], thus the composition of molecular features in our PDX pairs should be carefully addressed on levels of both single-cell and multi-omics. This study was hence designed to explore the mechanisms and overcoming strategies of acquired Trastuzumab resistance by single-cell transcriptome sequencing combined with profiling of proteomic and genomic variations, followed by phenotypical & mechanistic validation in preclinical PDX models.

## Results

### Establishment and identification of Trastuzumab acquired resistant PDX model in gastric cancer

**Fig. 1:**
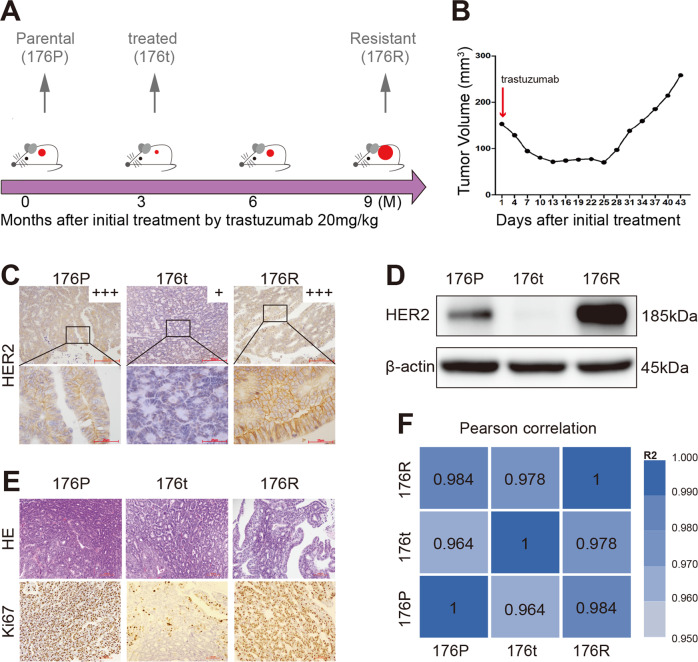
Establishment and identification of Trastuzumab acquired resistant PDX model in gastric cancer. **A** The establishment procedure of Trastuzumab acquired resistant PDX model. The mice of 176P were exposed to Trastuzumab at the dose of 20 mg/kg for about 9 months (5 passages) to obtain the resistant model 176R. **B** Tumor growth curve of the last passage when the model was established. **C** HER2 expression before and after Trastuzumab resistance identified by immunohistochemistry (x200 for the upper row, x1000 for the lower row). **D** HER2 expression before and after Trastuzumab resistance identified by western blot. **E** H&E (x200) and Ki67 (x200) staining showed the Lauren classification, histological differentiation, and cell proliferation (176P > 90%, 176t < 10%, 176R > 90%), respectively. **F** Pearson correlation coefficient (presented by R^2^ value) of samples by RNA-seq confirmed the tissue homology before and after Trastuzumab resistance.

A naïve PDX model (case 176) sensitive to Trastuzumab was selected from our PDX model library to establish the Trastuzumab acquired resistant PDX model (the clinicopathological characteristics of case 176 were shown in Table 1). This Trastuzumab sensitive PDX model was treated with Trastuzumab (20 mg/kg, intraperitoneal injection) for about 9 months (5 passages) to generate acquired resistance. Along with the process, samples were preserved according to clinical practice: 176P represented the naïve parental, 176t represented the naïve tumor being treated with Trastuzumab and displaying sensitivity to the drug, and 176R represented the acquired resistance to Trastuzumab (Fig. 1A, B). HER2 expression and Ki67 staining of tumors before and after Trastuzumab resistance (Fig. 1C–E) suggested the dynamic HER2 expression and cell proliferation with the response to Trastuzumab treatment. Moreover, the HE-based histological traits and RNA-based tumor homology were highly consistent before and after Trastuzumab resistance (Fig. 1E, F). Therefore, 176P and its derivative linage 176R represented a pair of GC PDX models suitable for the study of acquired Trastuzumab resistance.

**Table 1:** The clinicopathological characteristics of case 176.

Variable	Characteristics
Patient code	176
Gender	Male
Age at first diagnosis	60 y
HER2 status by IHC	Positive (3+)
Pathology	Adenocarcinoma
Lauren classification	Intestinal
Differentiation	Moderate

### Gene expressional changes, rather than genomic variations, contributed to acquired Trastuzumab resistance

**Fig. 2:**
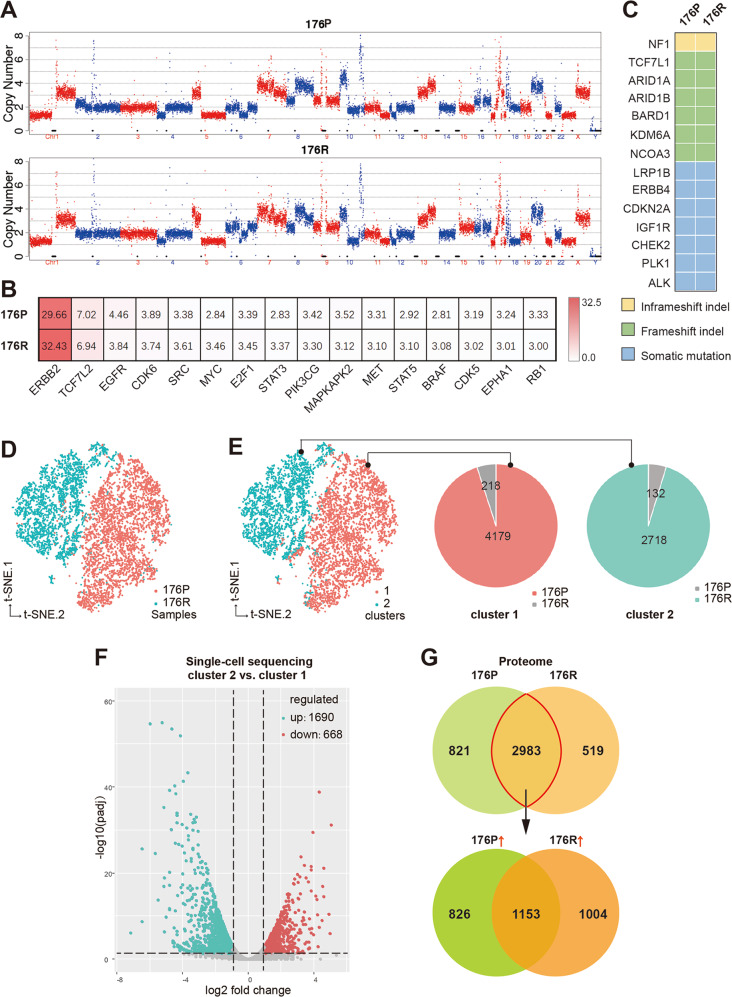
Gene expressional changes, rather than genomic variations, contributed to acquired Trastuzumab resistance. **A** The full view of whole chromosomal copy number in 176P and 176R tissues by whole-genome sequencing (WGS). **B** The copy number of some cancer-related genes in 176P and 176R tissues. **C** The mutation profiles of cancer-related genes were consistent in 176P and 176R tissues. **D** Cells derived from 176P and 176R tissues were well divided into two groups after t-SNE algorithm analysis by single-cell RNA sequencing. **E** According to the k-means clustering algorithm, all the cells were divided into two separated subgroups when *k* = 2. Cells derived from 176P and 176R tissues were dominant in cluster 1 (pink) and cluster 2 (green), respectively. **F** Volcano plot displayed differentially expressed molecules of cluster 1 and cluster 2 when fold change ≥2. **G** Venn diagram showed differentially expressed molecules in 176P and 176R tissues detected by protein mass spectrometry. A total of 2983 molecules expressed both in 176P and 176R tissues, among which 1004 molecules were upregulated after acquired Trastuzumab resistance when fold change ≥ 1.5.

Based on the whole-genomic analysis, no significant changes were observed addressing chromosomal copy number variation (Fig. 2A), critical gene amplification (including ERBB2, Supplementary Fig. S3), somatic mutation, and indel (insertion-deletion) between 176P and 176R tissues (Fig. 2B, C), suggesting no de novo variations involved in acquired Trastuzumab resistance.

By t-SNE algorithm of scRNA-seqencing data, cells derived from 176 P and 176R tissues were well divided into two groups, suggesting obvious gene differential transcriptional profiles between 176P and 176R tissues (Fig. 2D). Moreover, according to k-means clustering algorithm, the total 7247 cells were mapped into two robust clusters (cluster 1 and 2; Fig. 2E) when *k*-value was 2, displaying a very similar classification to the origination classification of 176P and 176R (Fig. 2D). According to gene expression profiles, cells derived from 176P tissue were dominant in cluster 1 (pink) while cells derived from 176R tissue were dominant in cluster 2 (green). Compared to cluster 1 that majorly containing 176P-derived cells, 1690 genes were upregulated in the 176R-dominant cluster 2 (Fig. 2F; fold change ≥2; *Padj* ≤ 0.05). On the other hand, proteomic analysis also showed numerous differentially expressed molecules between 176P and 176R (Fig. 2G). These result indicated that gene expressional changes, rather than genomic variations, contributed to acquired Trastuzumab resistance.

### Acquired Trastuzumab resistance was accompanied with lower intratumoral heterogeneity and differential cell phenotypes

Based on k-means clustering algorithm, as the k-value increased from 3 to 9, one or two dominant cell subpopulations remained in 176R tissue, while cells derived from 176P tissue could be classified to several clusters indicated as more cellular diversity (Supplementary Fig. S4A–D), suggesting that intratumoral heterogeneity decreased after acquired Trastuzumab resistance, which might be due to the pressure of screening under drug exposure.

**Fig. 3:**
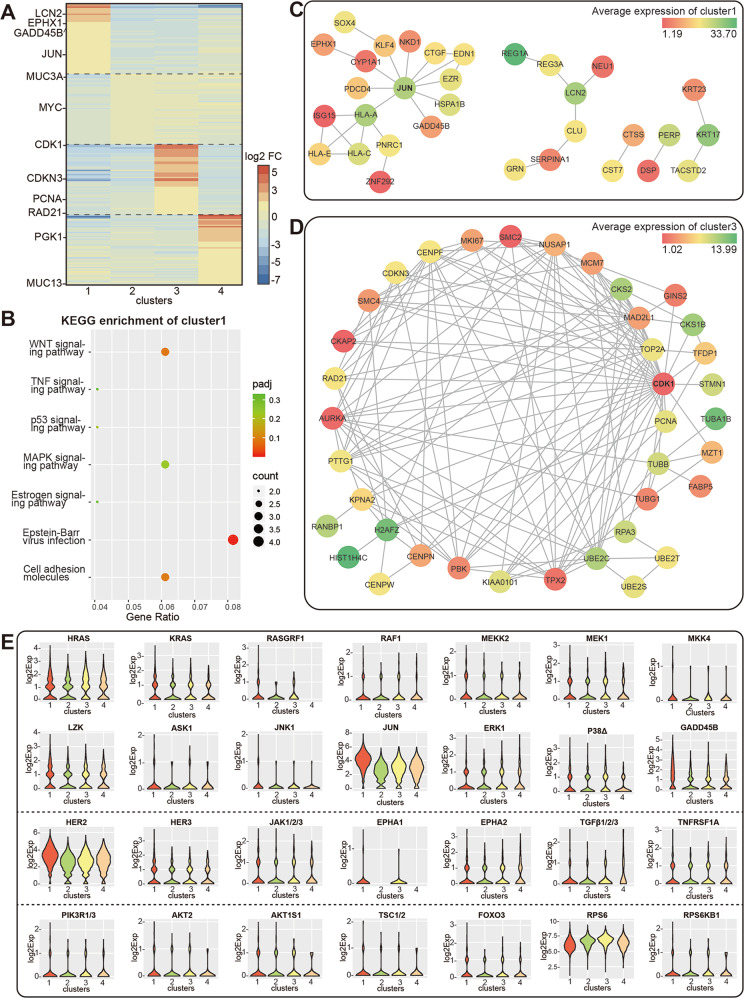
Intratumoral heterogeneity and differential cell phenotypes in 176P and 176R tissues. **A** Heatmap showed the top 50 upregulated genes in each cluster. The color bar represented the value of log2 fold change. **B** The top 50 genes of cluster 1 were enriched by KEGG database. **C**, **D** Network diagrams of the top 50 genes of cluster 1 (confidence Score = 0.400) and cluster 3 (confidence score = 0.900) according to STRING database. The color bar represented the average expression of genes in each cluster. **E** Violin plots showed the expression profile of some critical molecules that were involved in membrane receptor tyrosine kinase, MAPK, PI3K/AKT signaling pathways in four clusters.

According to Supplementary Fig. S4, when *k*-value was 4 or less (*k* ≤ 4), only one cell subpopulation was dominant in 176R tissue, and when *k*-value was 4 or more (*k* ≥ 4), cells derived from 176P tissue were divided into three major groups. Therefore, *k* = 4 was adopted to generate four public clusters to describe the major composition of 176R (cluster 1 = 86.3%) and 176P (cluster 2 + 3 + 4 = 98.4%) tissues. By outlining the top 50 upregulated genes in each cluster compared to the other 3 clusters (Fig. 3A), we found that the top 50 genes in cluster 1 were enriched by the KEGG database to signaling pathways such as WNT and MAPK (Fig. 3B), cluster 2 and 4 were enriched to transcription factor regulation, while cluster 3 was enriched to cell cycle, DNA replication and mismatch repair (Supplementary Fig. S5A). Furthermore, we analyzed the network diagrams of the top 50 genes in each cluster through applying STRING database (https://string-db.org/), and found that JUN and CDK1 were the hub genes in cluster 1 (Fig. 3C) and cluster 3 (Fig. 3D), as well as RPS3 in cluster 2 and LDHA, ALDOA, ALDOC in cluster 4 (Supplementary Fig. S5B–C), respectively. As described above, Supplementary Fig. S5D displayed the heatmap of differential genes related to cell biological process and signaling pathways by KEGG categories in four clusters. Also, the expression distribution of some critical molecules which might contribute to Trastuzumab resistance were presented by the violin plots in Fig. 3E and Supplementary Fig. S5E.

### Homology analysis revealed the evolution related with acquired Trastuzumab resistance

**Fig. 4:**
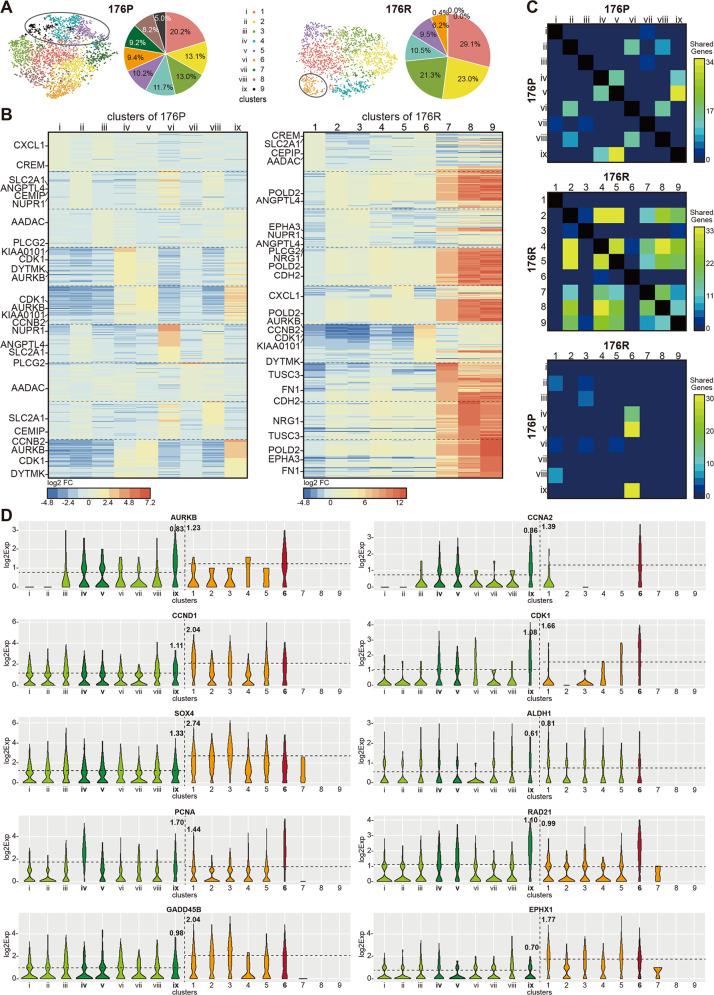
Homology analysis revealed the evolved process from parental tissue to Trastuzumab resistant tissue. **A** 4311 cells of 176P and 2936 cells of 176R were divided into nine clusters by k-means algorithm, respectively. **B** Heatmaps showed the top 50 upregulated genes in each cluster compared to other eight clusters of 176P and 176R tissues. **C** Shared genes between clusters in the same samples or between clusters in different samples. **D** Some differentially expressed shared genes in different clusters of 176P and 176R tissues. Cluster 6 and cluster ix, v, iv were highlighted. Dashed lines represented log2 average expression of cells in both samples.

To trace the origin of the phenotypic differences before and after Trastuzumab resistance, 4311 cells from 176P and 2936 cells from 176R were divided into 9 clusters by k-means algorithm respectively (Fig. 4A). Compared to 176P tissue, the intratumoral heterogeneity in 176 R tissue was decreased as indicated by fewer dominant clusters. The heatmaps in Fig. 4B showed the top 50 upregulated genes in each cluster that compared to the other eight clusters. As demonstrated by inter-model or intro-model comparisons (Fig. 4C), the shared genes across clusters dramatically increased from 176P to 176R, also suggesting a reduced intratumoral heterogeneity. Interestingly, cluster 6 of 176R tissue had more shared genes with clusters ix, v, and iv of 176P tissue, which were emphasized in Fig. 4A and were consistent with the result when *k*-value was 4 (Supplementary Fig. S6A–B). The violin plots showed some differentially expressed genes in different clusters of 176P and 176R tissues (Fig. 4D and Supplementary Fig. S6C–D), such as CCNA2, a member of the cyclin family involved in cell cycle and the functions of which deserved to be further elucidated.

### Alterations of tissue protein expression profile and phosphorylation profile after Trastuzumab resistance

**Fig. 5:**
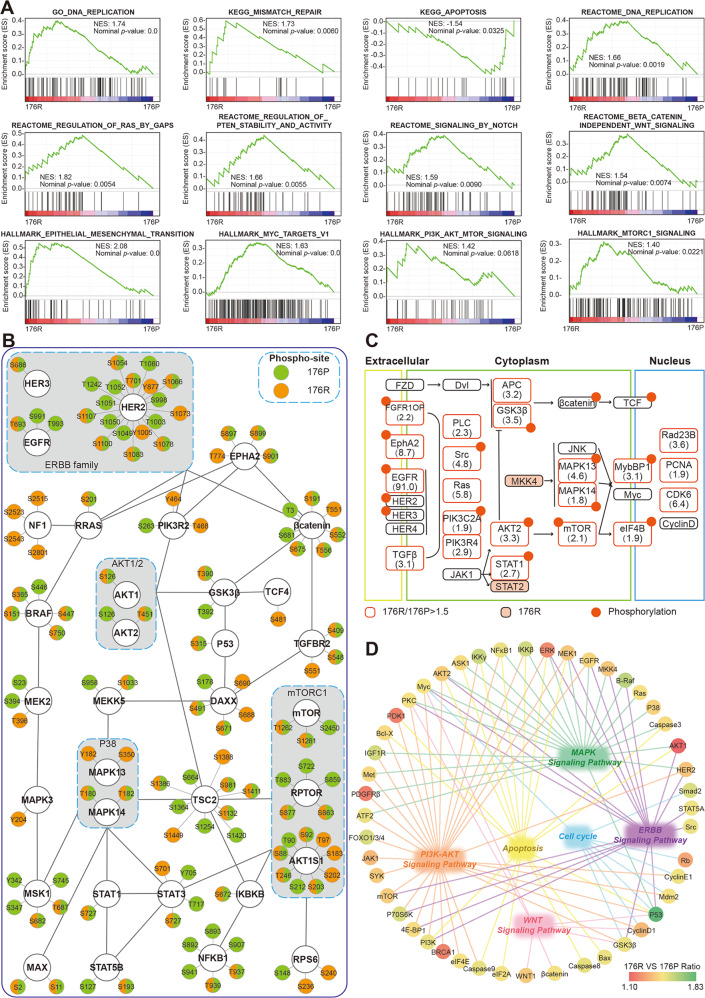
Protein expression profile and phosphorylation profile in tissues before and after Trastuzumab resistance. **A** The total 2983 proteins expressed both in 176P and 176R tissues were enriched according to GO, KEGG, REACTOME, and HALLMARK datasets, by GSEA. NES: Normalized Enrichment Score. **B** Phosphorylation profiles of some important molecules identified by protein chip. **C** The upregulated and phosphorylated proteins were distributed in various signaling pathways after Trastuzumab resistance by proteomic analysis. **D** The upregulated proteins found by protein chip were involved in several critical pathways including MAPK, PI3K/AKT, WNT pathways, and so on.

As previously described (Fig. 2G), our proteomic profiling identified a total of 2983 proteins expressed both in 176P and 176R tissues. Based on these molecules, a series of crucial cancer-related pathways were assessed with Gene Set Enrichment Analysis (GSEA). Except for apoptosis, other signaling pathways including DNA replication, mismatch repair, cell cycle, epithelial-mesenchymal transation, RAS, PTEN, NOTCH, WNT, MYC, MTOR were significantly enriched in 176R tissue (Fig. 5A and Supplementary Fig. S7A), suggesting their involvement in acquired Trastuzumab resistance. For 1004 molecules upregulated in 176R tissue, several pathways including MAPK, mismatch repair, cell cycle, etc. were also enriched by KEGG pathway enrichment (Supplementary Fig. S7B).

Furthermore, based on the phosphorylation profiles analyzed by protein chips (Fig. 5B and Supplementary Fig. S7C) almost all molecules had distinct phosphorylation patterns between 176P and 176R tissues, suggesting their expression changes after developing Trastuzumab resistance. It worth to be mentioned that phosphorylated NF1 was only detected in 176R tissue, which negatively regulates MAPK cascade and contributed to Trastuzumab resistance. The key signaling nodes with upregulated expression/phosphorylation after Trastuzumab resistance (176R/176P fold change ≥ 1.5) were identified (Fig. 5C). Moreover, the molecules upregulated in 176R tissue found by protein chips were mainly involved in several critical pathways including MAPK, PI3K/AKT, WNT pathways (Fig. 5D and Supplementary Fig. S8).

### Validation for critical molecules and signaling pathways involved in acquired Trastuzumab resistance

**Fig. 6:**
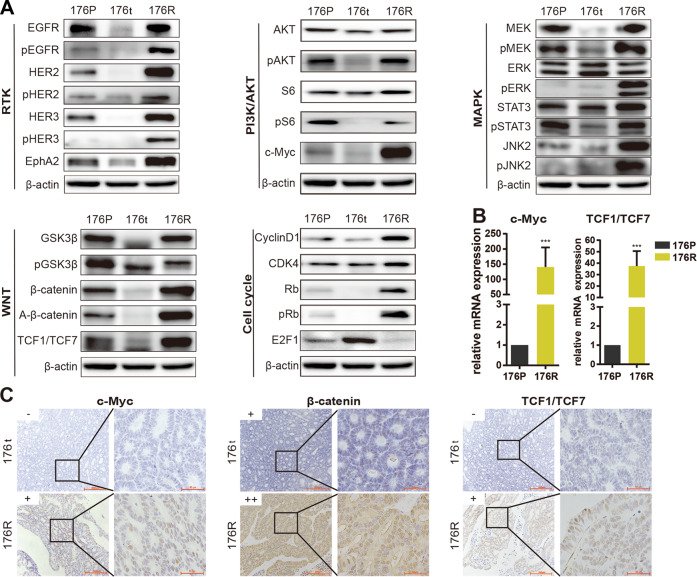
Multiple critical molecules and signaling pathways were involved in Trastuzumab resistance. **A** The expressions of several membrane receptors and critical molecules of PI3K/AKT, MAPK, WNT, and cell cycle pathways were detected by western blot. β-actin as endogenous control; RTK, receptor tyrosine kinase. A-β-catenin, non-phospho β-catenin at Ser45. **B** The mRNA expressions of transcription factors c-Myc and TCF1/TCF7 were measured using quantitative PCR. *p < 0.05, **p < 0.01, ***p < 0.001. **C** The nuclear expressions of c-Myc, TCF1/TCF7 and β-catenin were evaluated by immunohistochemistry (IHC, x200 for the left row, x1000 for the right row). The staining score of IHC was defined as 0, 1+, 2+, and 3+.

We then performed experimental investigations in 176R/176P PDX models to verify the pathway changes identified by multi-omics study. As expected, expression and activate phosphorylation of ERBB family members (including EGFR, HER2, HER3) or partner (EphA2) were significantly reduced upon sensitive Trastuzumab response and upregulated after developing Trastuzumab resistance (Fig. 6A, Supplementary Fig. S10). On the other hand, several major downstream signaling pathways were reactivated in 176R tissue, including PI3K/AKT-MAPK signaling marked by expression or phosphorylation of AKT/S6/MEK/ERK/STAT3/JNK2/c-Myc, WNT/β-catenin signaling marked by expression or phosphorylation of β-catenin/activated-β-catenin/GSK3β/TCF1/TCF7, cell cycle signaling marked by expression or phosphorylation of cyclin D1/CDK4/Rb/E2F1 (Fig. 6A–C). Our data suggested the activation of these pathways contributed to acquired Trastuzumab resistance, which emphasized the complexity of resistance mechanisms.

### Trastuzumab combined with HER3-targeted antibody or MEK inhibitor were promising strategies for overcoming acquired Trastuzumab resistance

**Fig. 7:**
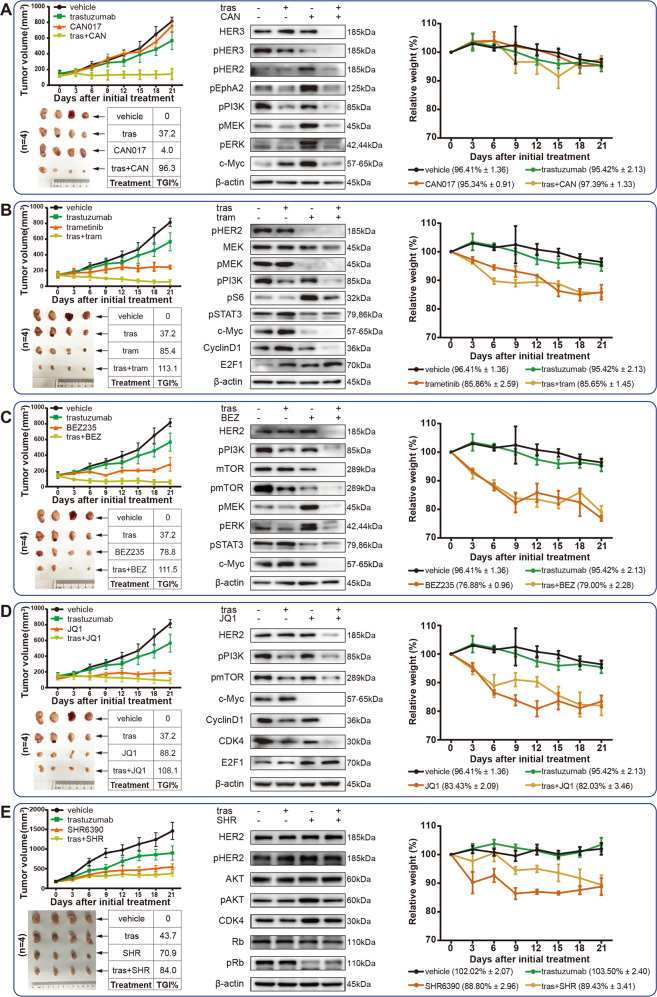
The antitumor efficacy of several inhibitors alone or in combination with Trastuzumab in Trastuzumab resistant model 176 R. Mice (*n* = 4 per group) were treated with vehicle, Trastuzumab alone, inhibitors alone, and inhibitor combined with Trastuzumab for 21 days. (**A** CAN017; **B** trametinib; **C** BEZ235; **D** JQ1; **E** SHR6390). Tumor volume was calculated and tumor growth curve was plotted, as well as the TGI and relative weight of the initial treatment. The related protein expressions were also displayed in each figure. TGI tumor growth inhibition, tras Trastuzumab, CAN CAN017, tram trametinib, BEZ BEZ235, SHR SHR6390.

Combined therapeutic strategies were well confirmed to be powerful options to overcoming acquired resistance. Since the activation of several pathways contributed to acquired Trastuzumab resistance, we assessed the validity of their corresponding inhibitors, including HER3-targeted antibody (CAN017), MEK inhibitor (trametinib), PI3K/mTOR inhibitor (BEZ235), c-Myc inhibitor (JQ1), and CDK4/6 inhibitor (SHR6390), in overcoming acquired Trastuzumab resistance. CAN017 alone had no antitumor effect at all, yet its combination with Trastuzumab exhibited a powerful tumor growth inhibition (TGI) of 96.3% (Fig. 7A). Consistent with the antitumor activity, the expressions of HER3, HER2 and their downstream signaling pathway were inhibited by CAN017 combined with Trastuzumab (Fig. 7A). Unlike CAN017, other inhibitors (trametinib, BEZ235, JQ1, and SHR6390) alone showed certain TGIs ranged from 70.9% to 88.2%, and their combination with Trastuzumab displayed TGIs ranged from 84% to 113.1% (Fig. 7B–E). Similarly, the related targets and their downstream pathways were also inhibited under each inhibitor combined with Trastuzumab (Fig. 7B–E). WB-quantified changes of these pathways were displayed by Supplementary Fig. S11.

However, although the combination strategies had achieved a satisfactory antitumor effect, the tolerance of each drug also needed to be considered. The mice tolerated CAN017 and trametinib well with less weight loss, yet other inhibitors induced significant weight loss (Fig. 7, Supplementary Table S3). By collectively considering both antitumor effects and drug tolerances, Trastuzumab combined with HER3-targeted antibody or MEK inhibitor were promising strategies for overcoming acquired Trastuzumab resistance.

## Discussion

As the first-line option of targeted therapy for HER2-positive GC, Trastuzumab significantly improves patient survival and has opened the era of precision medicine in GC. However, the acquired resistance remains to be a long-standing problem, while satisfying second-line targeted therapy remains unelucidated. The high heterogeneity of GC resulted in the complexity of molecular constitution as well as the lack of best preclinical research models, as a consequence, the exact molecular mechanism of Trastuzumab resistance in GC patients has not yet been fully figured out so far.

Patient-derived xenograft (PDX) models are the best choice for preclinical study at present due to their high fidelity with patient characteristics. According to current reports, Trastuzumab sensitive GC PDX model and its paired PDX model with acquired Trastuzumab resistance were rare. In this study, we successfully established such a pair of Trastuzumab-resistant models, then performed multi-omics study to explore the resistance mechanisms and overcoming strategies after Trastuzumab resistance. Some studies reported that genetic variations of PIK3CA, ERBB2/4, and NF1 might contributed to Trastuzumab resistance [15], however, we found that instead of genetic variations, it were expressional changes contributed to acquired Trastuzumab resistance in our pair of PDX models.

Many differentially expressed molecules were found between 176P and 176R tissues based on multi-omics analysis, suggesting cell phenotypes were altered after Trastuzumab resistance. According to single-cell sequencing, phenotypes of the cells in 176R tissue were more concentrated than those in 176P tissue, suggesting the decreased intratumoral heterogeneity after Trastuzumab resistance, which might be due to the screening under drug exposure. Although divergence exist for the differentially expressed molecules identified on RNA and proteins levels, several critical molecules and pathways including HER3, EphA2, PI3K/AKT, MAPK pathway, WNT pathway, and cell cycle, were highlighted and validated in vitro (Fig. 6), while other molecules potentially involved in Trastuzumab resistance needed to be further validated.

Besides the activation of certain oncogenic pathways, it was well known that cancer stem cells were also a main cause inducing drug resistance [25]. We also validated several stemness-related markers including CD44, CD133, Nanog, and SOX2, yet no significant changes were found between 176P and 176R tissues (Supplementary Fig. S9A). In addition, based our preliminary results, DNA repair and EMT (epithelial-mesenchymal transition) might be involved in Trastuzumab resistance (Fig. 5A), which were also evaluated in vitro. As a key molecule involved in DNA repair, c-PARP (the cleaved active form of PARP) was inhibited and RAD51 was upregulated after Trastuzumab resistance, suggesting DNA repair abnormality might contributed to Trastuzumab resistance (Supplementary Fig. S9A). For EMT process, several markers including E-cadherin, vimentin, Snail, and Claudin 1 were evaluated and only vimentin obviously increased in 176R tissue compared to 176P tissue (Supplementary Fig. S9A).

Based on bioinformatics explorations, we verified the overcoming strategies of Trastuzumab resistance. As shown by in vivo data, except Trastuzumab and CAN017 alone, other single drugs or combination treatments displayed a certain degree of tumor inhibition, among which Trastuzumab combined with HER3-targeted antibody or MEK inhibitor demonstrated both high efficacy and good tolerance, suggesting a quite promising strategy for future clinical trials. Although the antitumor activity of Trastuzumab combined with CDK4/6 inhibitor was slightly lower than other combinative strategies, this strategy also worth to be validated by clinical trials. In our recent report, we verified the strong synergistic tumor inhibition of pyrotinib (a novel small-molecule tyrosine kinase inhibitor mainly targeting HER2) combined with CDK4/6 inhibitor in HER2-positive GC patients [26]. For Trastuzumab combined with PI3K/mTOR inhibitor or c-Myc inhibitor, considering its very good efficacy, further investigations were worth to be conducted by adjusting the drug doses to achieve higher efficiency and lower toxicity.

In summary, we successfully established a homologous pair of GC PDX models reflecting acquired Trastuzumab resistance, and comprehensively delineated the landscape of Trastuzumab resistance mechanisms using multi-omics analysis. Also, our results provided reliable evidence for future clinical trials to validate the promising strategies for overcoming Trastuzumab resistance.

## Materials and methods

### Animal experiments

All animal experiments were carried out with 5-week-old, 18–25 g NOD/SCID female mice at SPF (Specified Pathogen Free) condition under the approval of the Institutional Ethics Committee of Peking University Cancer Hospital & Institute. The procedures of PDX model establishment and drug administration were described according to our previous reports [18, 26, 27]. When tumor volume reached 150–200 mm^3^, mice were randomized into different groups followed by drug administration (5 mice per group). Tumor volume and body weight were measured every other day with vernier calipers.

Drug administration in animal experiments were as follows: vehicle (normal saline), daily gavage or weekly intraperitoneal injection for 3 weeks; Trastuzumab (Roche), 20 mg/kg, weekly intraperitoneal injection for 3 weeks; CAN017 (CANbridge), 20 mg/kg, twice a week intraperitoneal injection for 3 weeks; BEZ235 (Selleck), 50 mg/kg, daily gavage for 3 weeks; trametinib (Selleck), 1 mg/kg, daily gavage for 3 weeks; JQ1 (Selleck), 50 mg/kg, daily intraperitoneal injection for 3 weeks; SHR6390 (Hengrui Medicine), daily gavage for 3 weeks. Tumor size and body weight of mice were measured every 3 days. Tumor volume (V) and tumor growth inhibition (TGI) were calculated by the following formula, respectively.

$$V = {\frac{W^2L}2}$$

(*V:* volume*, W:* width of tumor short diameter*, L:* length of tumor long diameter)

$${\rm{TGI}}\, = \,\left(1 - \frac{{{\it{\Delta }}M}}{{{\it{\Delta }}C}}\right)\, \times \,{\it{\rm{100}\% }}$$

(Δ*M* = tumor volume changes of the medication group, Δ*C* = tumor volume changes of the control group on the last day of the study)

### Genomic DNA and total RNA extraction for high-throughput sequencing

Genomic DNA and total RNA from PDX tissue samples were extracted by a QIAamp DNA Mini Kit (Qiagen, Hilden, Germany) and TRIzol reagent (#15596018, Invitrogen, USA), respectively. All samples were sequenced on a HiSeq4000 platform (Illumina, USA) with about 5 million paired-end reads and the average length of each read was 100 bp per sample. Sequence data that passed quality control (QC) was used for copy number variation (CNV), single-nucleotide variant (SNV) and RNA expression level analysis by low-coverage whole-genome sequencing (LC WGS), targeted DNA sequencing and RNA-seq. The standard of QC measurement and more detailed protocols were performed as described previously [28–31]. The 483 gene-panel included in targeted DNA sequencing was listed in Supplementary Table S1, and the flow chat of omics analysis was shown in Supplementary Fig. S1.

### Single-cell transcriptome sequencing

Single-cell transcriptome sequencing (scRNA-seq) was performed using 10x genomics platform (CapitalBio Corporation, Beijing China) and was shown in Supplementary Figs. S1 and S2. Briefly, fresh 176 P and 176 R tumor tissues isolated from mice were rapidly shredded and digested with collagenase to prepare single-cell suspension followed by cell count and cell viability evaluation by Countess II Automated Cell Counter. Single-cell suspension with cell viability higher than 80% and cell concentration up to 1000 cells/μL was eligible for subsequent sequencing. Mixture of single-cell suspension and gel beads containing barcode was wrapped by oil droplets to produce GEMs (Gel Bead-In-EMulsions). Each droplet was labeled by a barcode, and barcodes were ranked by their Unique Molecular Identifier (UMI) counts in descending order (Supplementary Fig. S2A). A total of 7247 effective cells (4311 cells from 176P and 2936 cells from 176R) was assessed and sequenced reads from 7247 cells were analyzed by the computer algorithm. The generated gene-barcode matrices were analyzed by PCA (principal component analysis) and t-SNE (t-distributed stochastic neighbor embedding) dimensionality reduction that enabled each cell to obtain the horizontal and vertical coordinates of the two-dimension. Afterwards cell subpopulations were clustered by k-means algorithm to deeply comprehend the tumor heterogeneity which is critical to drug resistance. The violin plot in Supplementary Fig. S2B showed the distribution of gene number detected in 7247 cells and individual cell sequencing was performed with Illumina HiSeq platform.

### Protein profiling

Protein profiling analysis was accomplished by National Center for Protein Science, Beijing, China. Tumor tissues were minced and lysed in lysis buffer containing protease and phosphatase inhibitors followed by sonication. Protein lysate was collected and digested using the filter-aided sample preparation (FASP) method with trypsin for generation of tryptic peptides. After the separation, elution and drying, the tryptic peptides were analyzed by liquid chromatography tandem mass spectrometry (LC-MS/MS) on Orbitrap Fusion Lumos (Thermo Fisher Scientific, Rockford, IL, USA). All data were obtained using the Xcalibur software and protein quantification was calculated using a label-free, intensity-based absolute quantification (iBAQ) method. More detailed information about protein identification, quantification, and functional analysis were referred as our previous study [32].

### Protein chip

Protein chip was purchased from Full Moon BioSystems, USA, which was treated with blocking buffer in advance and then coincubated with biotin-labeled protein samples for 2 h at room temperature, followed by the incubation with Cy3-Streptavidin Solution for 20 min at room temperature in dark. After washed with deionized water, chips were scanned by Agilent SureScan Dx Microarray Scanner at 532 nm spectrophotometry. The protein expression ratio of the samples was calculated as follows:

$${\it{\rm{Protein}}} - \rm{expression}\,\rm{ratio} = \frac{{{\it{\rm{protein}}}\,{\it{\rm{expression}}}}}{{{\it{\rm{internal}}}\,{\it{\rm{reference}}}}}$$

### Real-time quantitative PCR

Primer 5.0 software was used to design the primers. c-Myc forward primer: 5′-TCCTGTACCTCGTCCGATTC-3′, c-Myc reverse primer: 5′-GGTTTGCCTCTTCTCCACAG-3′; TCF7 forward primer: 5′-AGCACCAAGAATCCACCACA-3′, TCF7 reverse primer: 5′-GATTCCCACCACTTGGAGCA-3′; β-actin forward primer: 5′-GAGCTACGAGCTGCCTGACG-3′, β-actin reverse primer: 5′-CCTAGAAGCATTTGCGGTGG-3′. The extracted RNAs from 176P and 176R tissues were reversed to cDNAs followed by evaluating the expression level of target gene using TranStart Top Green qPCR SuperMix kit (TransGen Biotech, Beijing, China) based on the ABI 7500 real-time PCR system (Applied Biosystems). The expression of target gene was evaluated by the comparative Ct (^ΔΔ^CT) method as the following formula.

$$^{\Delta \Delta }CT = \left( {CT_{tg}-CT_{rg}} \right)_{test}-\left( {CT_{tg}-CT_{rg}} \right)_{control}$$

*(tg: target gene, rg: reference gene)*

### Western blot

The total protein was extracted from tumor tissues with RIPA Lysis Buffer and protein concentration was measured with BCA Protein Assay Kit (Beyotime Biotechnology, Jiangsu, China). 30–50 μg of protein was separated on 10% SDS-PAGE gel, then transferred to nitrocellulose membranes. Nitrocellulose membrane was incubated with the corresponding primary antibodies at 4 °C overnight and secondary antibody at room temperature for 1 h. The proteins were detected by chemiluminescence and visualized by Amersham Imager 600. Grayscale analysis were conducted by ImageJ software. All antibodies used for western blot were listed in Supplementary Table S2.

### Immunohistochemistry (IHC) / hematoxylin and eosin (H&E) staining

Fresh tumor tissues were rapidly fixed with 10% formalin to prepare formalin-fixed paraffin-embedded (FFPE) blocks. FFPE slides were deparaffinized with xylene, hydrated with gradient ethanol, heated for antigen retrieval, treated with 3% H_2_O_2_ to remove endogenous peroxidase and blocked with 5% BSA, then incubated with primary antibodies overnight at 4 °C and secondary antibodies for 1 h at room temperature in turn. Finally, slides were stained with horseradish peroxidase (HRP) and diaminobenzidine substrate, followed by the score evaluation by two independent professional pathologists of Peking University Cancer Hospital. Ki67 index was calculated and staining score of IHC was defined as 0, 1+, 2+, and 3+. All antibodies used for IHC were listed in Supplementary Table S2.

### Data analysis

The experimental data were presented as mean ± SD, and statistical analysis was performed using SPSS 21.0 and GraphPad Prism 7.0. The differences of molecular expression between samples were analyzed with unpaired two-tailed *t* test or one-way ANOVA. *P* < 0.05 was considered as statistically significant, while adjusted *p* value (*P*adj) was applied in RNA-seq and single-cell sequencing. Annotation was performed on DAVID (https://david.ncifcrf.gov/tools.jsp). Gene/protein interaction information was obtained from STRING database (https://string-db.org/) and KEGG database (https://www.kegg.jp/kegg/). The network diagrams were then generated by R 3.5.1, Cytoscape 3.7.2 or Adobe Illustrator CC 2018. Gene set enrichment analysis (GSEA) was performed using GSEA 4.1.0 (https://www.gsea-msigdb.org/gsea/index.jsp).

## Supplementary information

uncropped WB1

uncropped WB2

Supplementary Figures & Tables

## Data Availability

All data relevant to the study are included in the article or uploaded as online supplemental information.
