# Supplementary figures and images for: Targeting HER3 or MEK overcomes acquired Trastuzumab resistance in HER2-positive gastric cancer-derived xenograft
Source: Cell Death Discov. 2022 Dec 3;8:478. doi: 10.1038/s41420-022-01259-z (PMC9719506; doi:10.1038/s41420-022-01259-z)

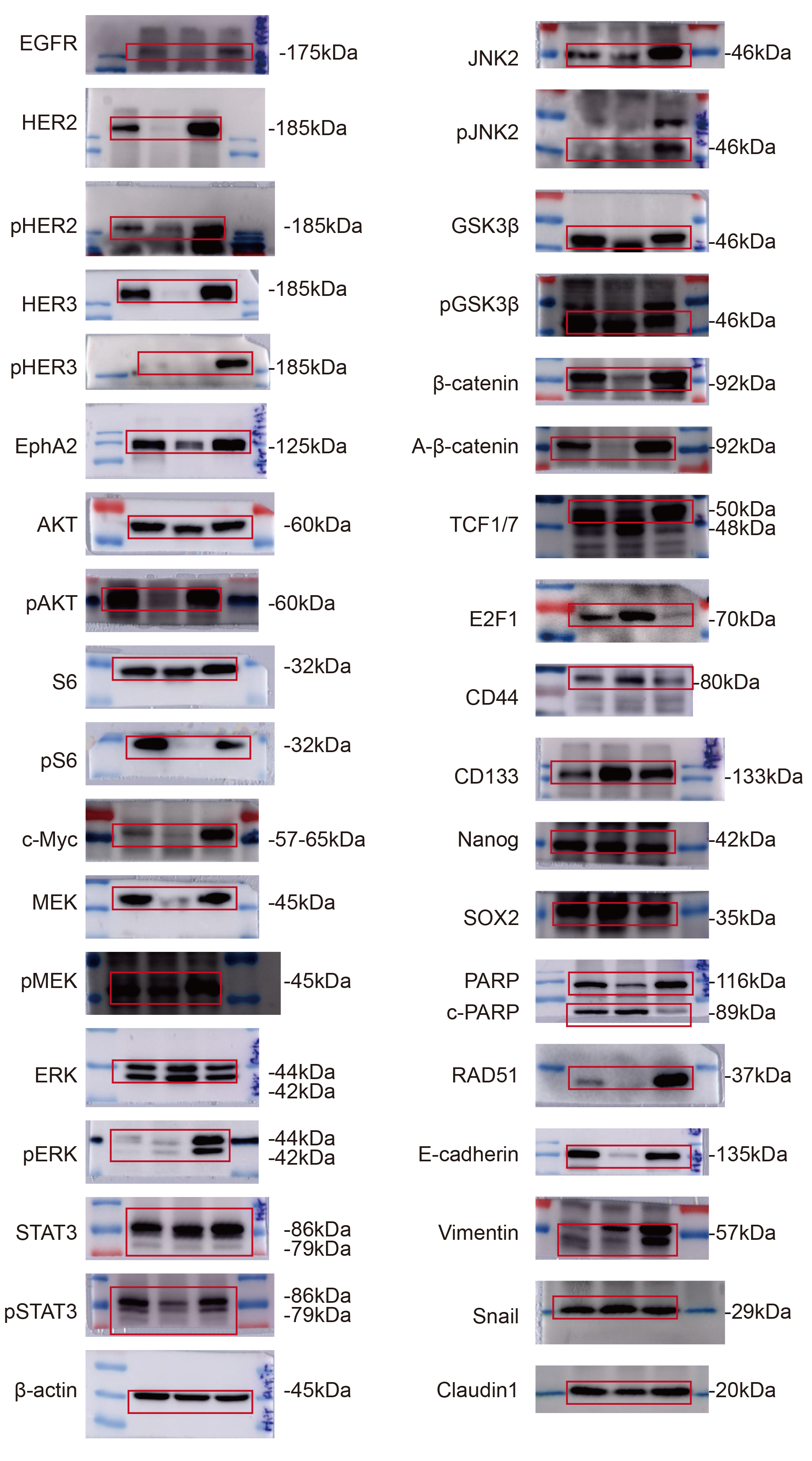

Supplement: Supplementary file 1 — uncropped WB1 [file 41420_2022_1259_MOESM1_ESM.tif]

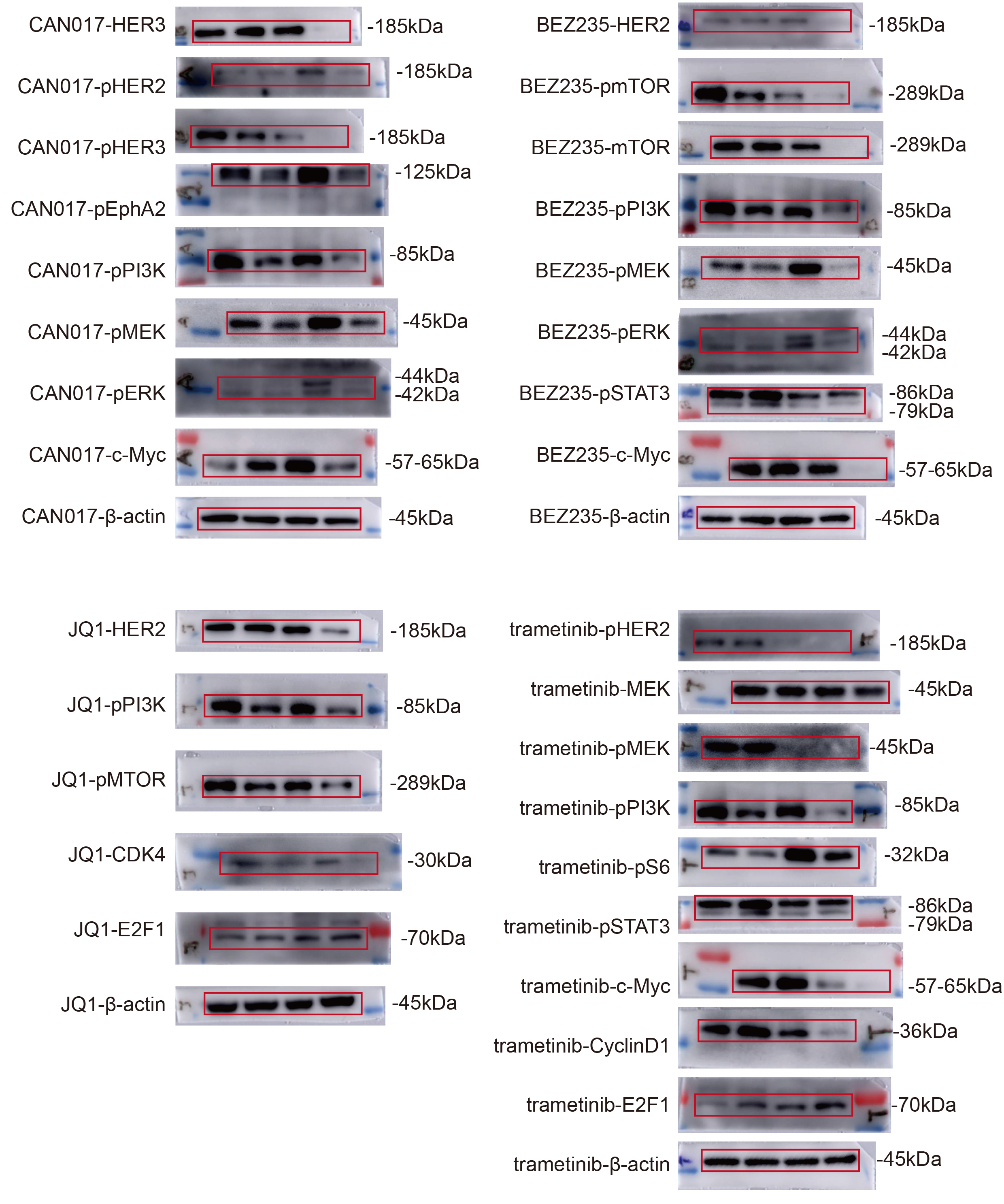

Supplement: Supplementary file 2 — uncropped WB2 [file 41420_2022_1259_MOESM2_ESM.tif]
