# Supplementary material for: Targeting HER3 or MEK overcomes acquired Trastuzumab resistance in HER2-positive gastric cancer-derived xenograft
Source: Cell Death Discov. 2022 Dec 3;8:478. doi: 10.1038/s41420-022-01259-z (PMC9719506; doi:10.1038/s41420-022-01259-z)
Supplement: Supplementary file 3 — Supplementary Figures & Tables [file 41420_2022_1259_MOESM3_ESM.docx]

**Supplemantary Figures & Supplemantary Figure Legends**

**
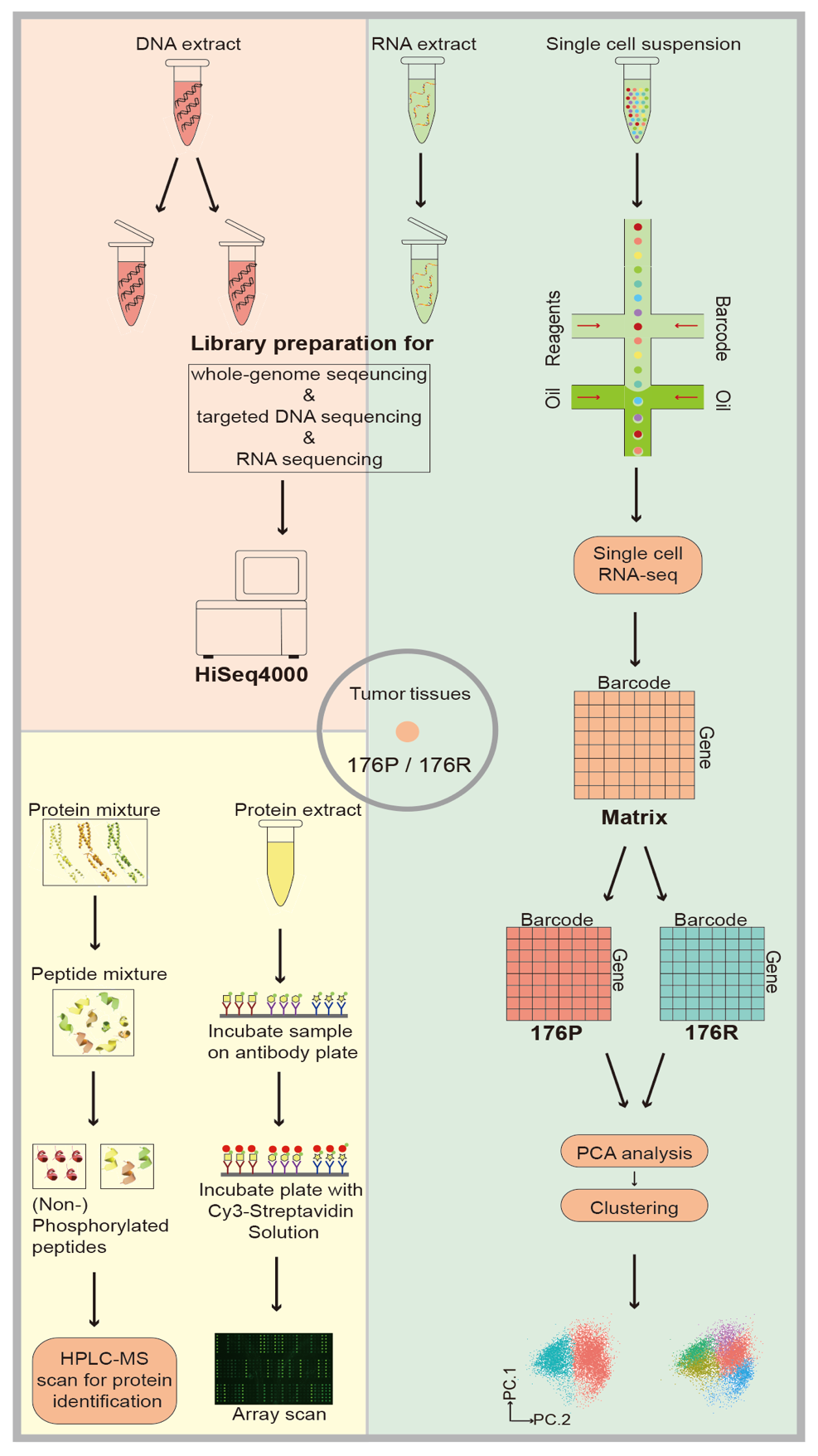
**

**Supplementary Figure S1. The flow chart of multi-omics analysis of tissues before and after trastuzumab resistance.** DNA and RNA extracted from 176P and 176R tissues were used for high-throughput sequencing, single cell suspensions were prepared for single-cell RNA sequencing and protein expression was detected by protein profiling and protein chip.


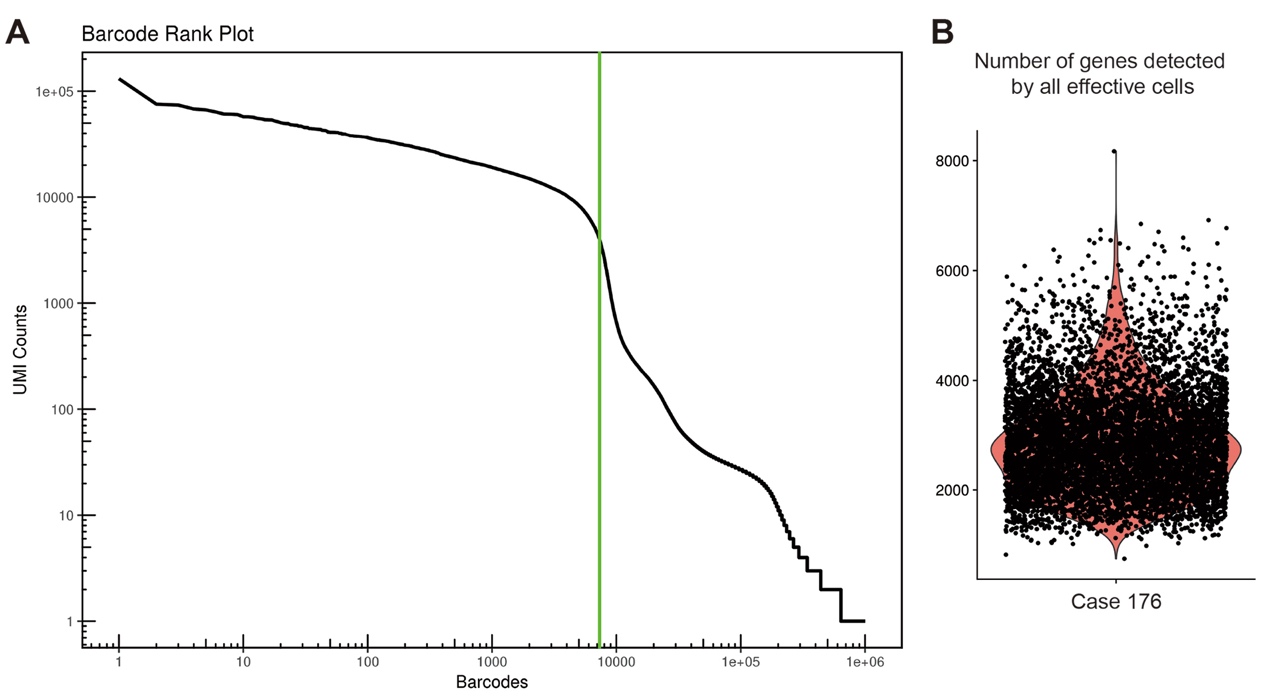


**Supplementary Figure S2. Evaluation of effective cells for single-cell transcriptome sequencing.** A. Barcodes were ranked according to the UMI (Unique Molecular Identifier) counts in descending order. The X-axis was Barcodes, and the left of the green line was the effective cells. B. Distribution of gene number detected by all the effective cells.


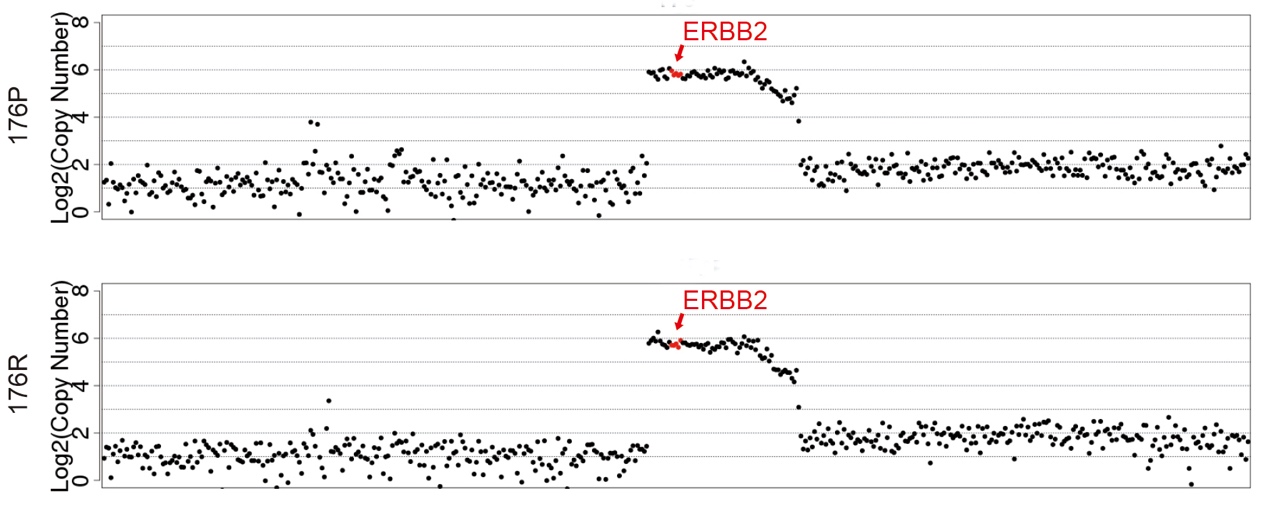


**Supplementary Figure S3. WGS showed ERBB2 amplification status of 176P and 176R.** Copy number was 43.69 of 176P and 48.14 of 176R.


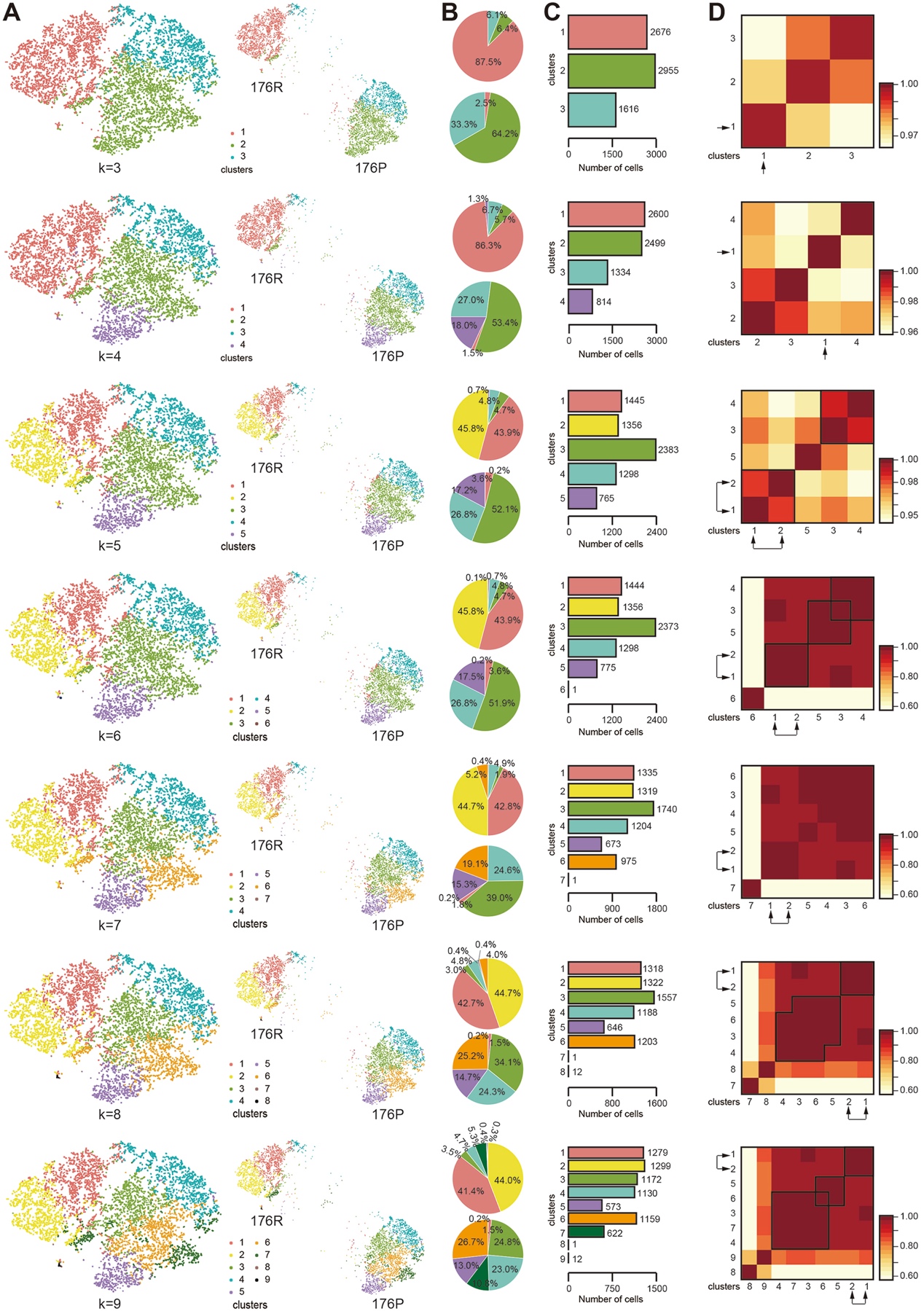


**Supplementary Figure S4. Cell subpopulation distributions of all cells when k=3-9 from top to the bottom.** **A.** 176P and 176R tissues had different dominant subgroups and few overlaps were existed. **B.** The proportion of each cluster in the two samples by pie charts showing fewer dominant subgroups in 176R than 176P tissues. **C.** Number of cells in each cluster of all cells when k=3-9 by bar graphs. **D.** Subpopulation correlations were represented by heat maps. The arrows indicated the dominant subgroups in 176R tissue.


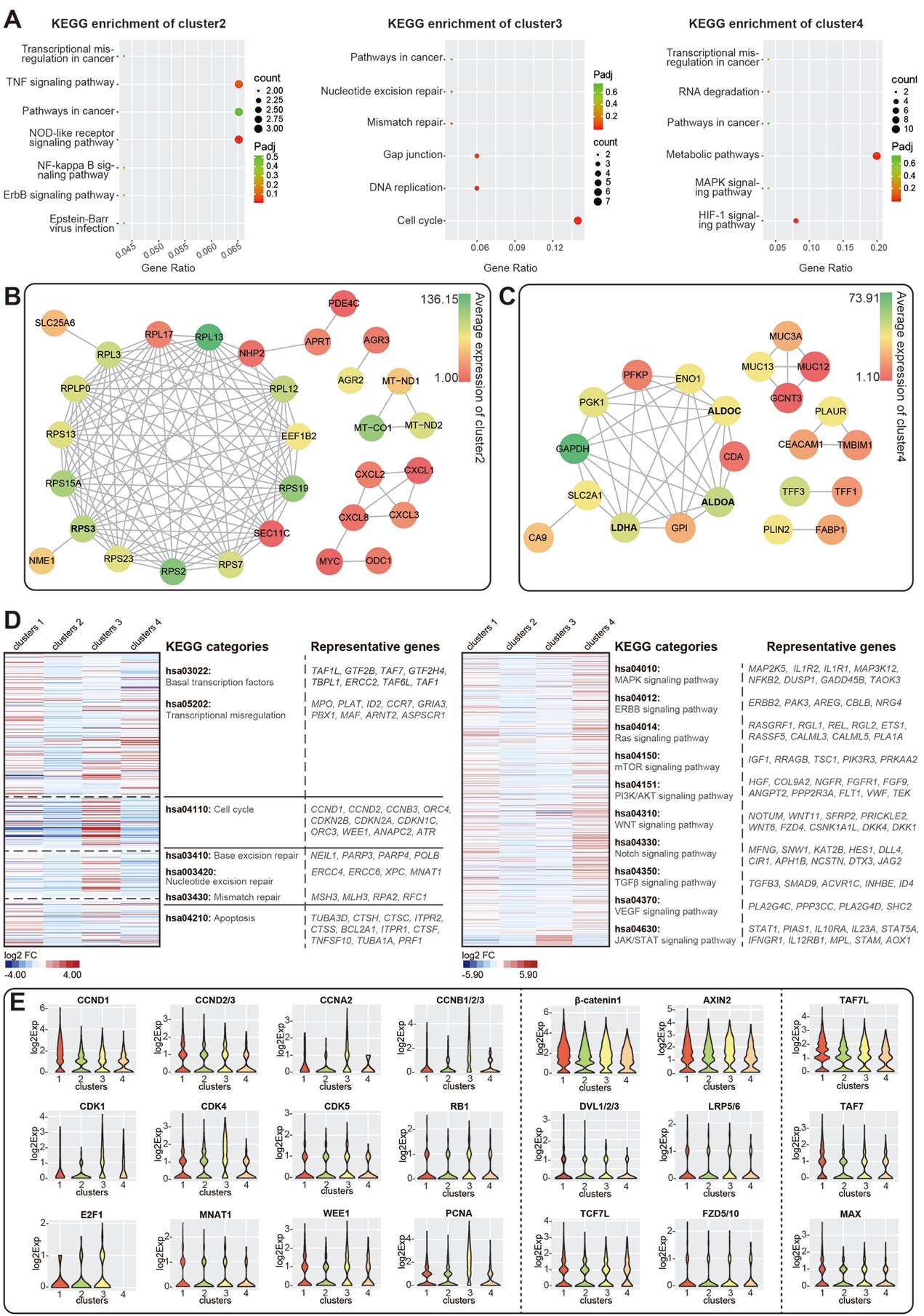


**Supplementary Figure S5. Different phenotypes in four clusters by scRNA-seq when k=4. A.** KEGG enrichment of top 50 upregulated genes in cluster 2, 3, and 4. **B, C.** Network diagrams of top 50 upregulated genes of cluster 2 (confidence score=0.700) and cluster 4 (confidence score=0.700) according to STRING database. The color bar represented the average expression of genes in each cluster. **D.** The heatmaps of genes related to multiple pathways according to KEGG categories with the hsa codes showed beside. E. Violin plots showed the expression profiles of some critical molecules that were involved in cell cycle, transcription factors, and WNT signaling pathways in four clusters.

**
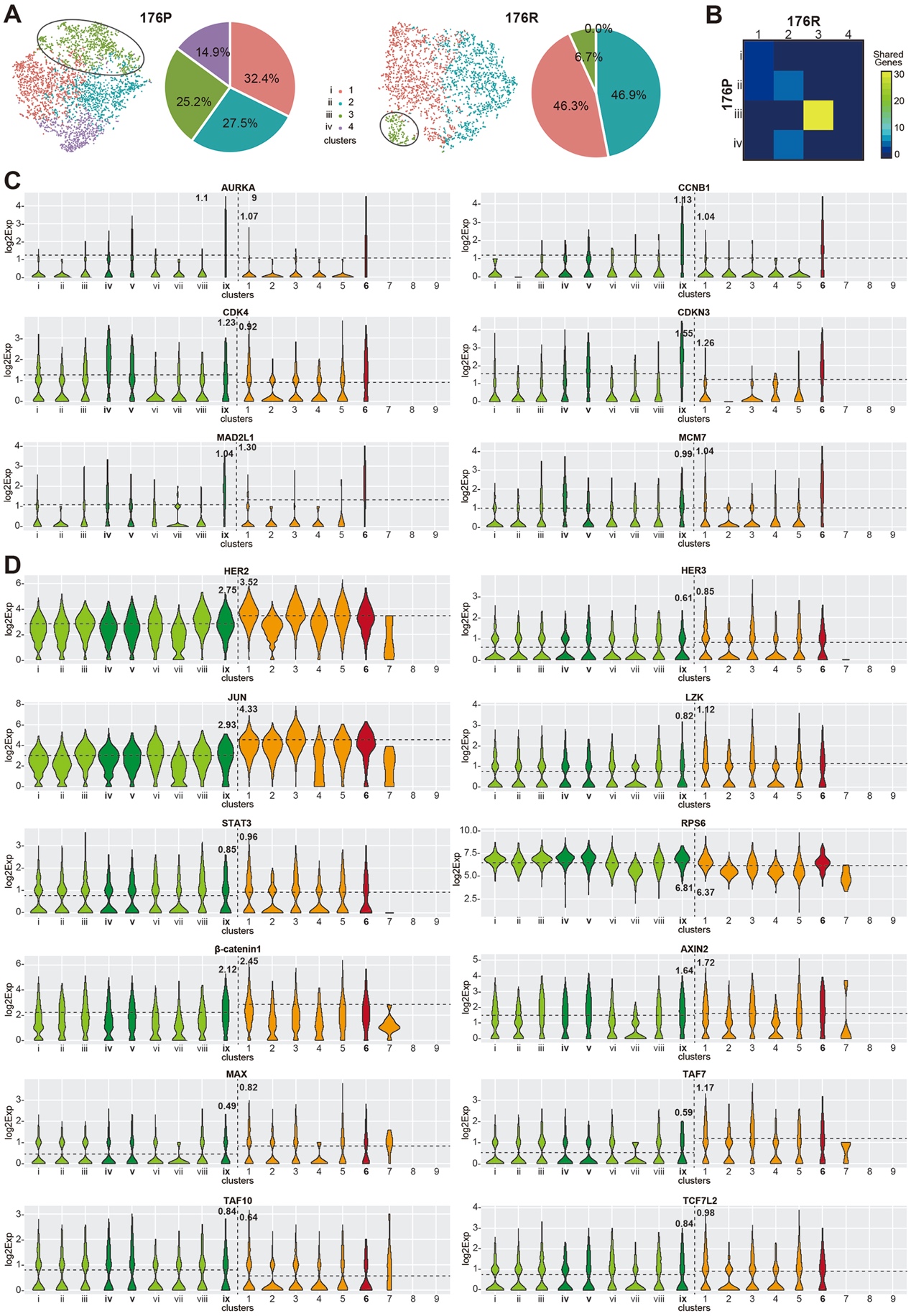
**

**Supplementary Figure S6. Homology analysis revealed the evolved process from parental tissue to trastuzumab resistant tissue. A.** 4311 cells of 176P and 2936 cells of 176R were divided into 4 clusters by k means algorithm, respectively. **B.** Shared genes between clusters of 176P and 176R tissues. **C.** Violin plots showed the expressions of partial top 50 genes. Cluster 6 and cluster ix, v, iv were highlighted. Dashed lines represented log2 average expression of cells in both samples. **D.** Violin plots showed the expressions of some enriched genes in previous results. Cluster 6 and cluster ix, v, iv were highlighted. Dashed lines represented log2 average expression of cells in both samples.

**
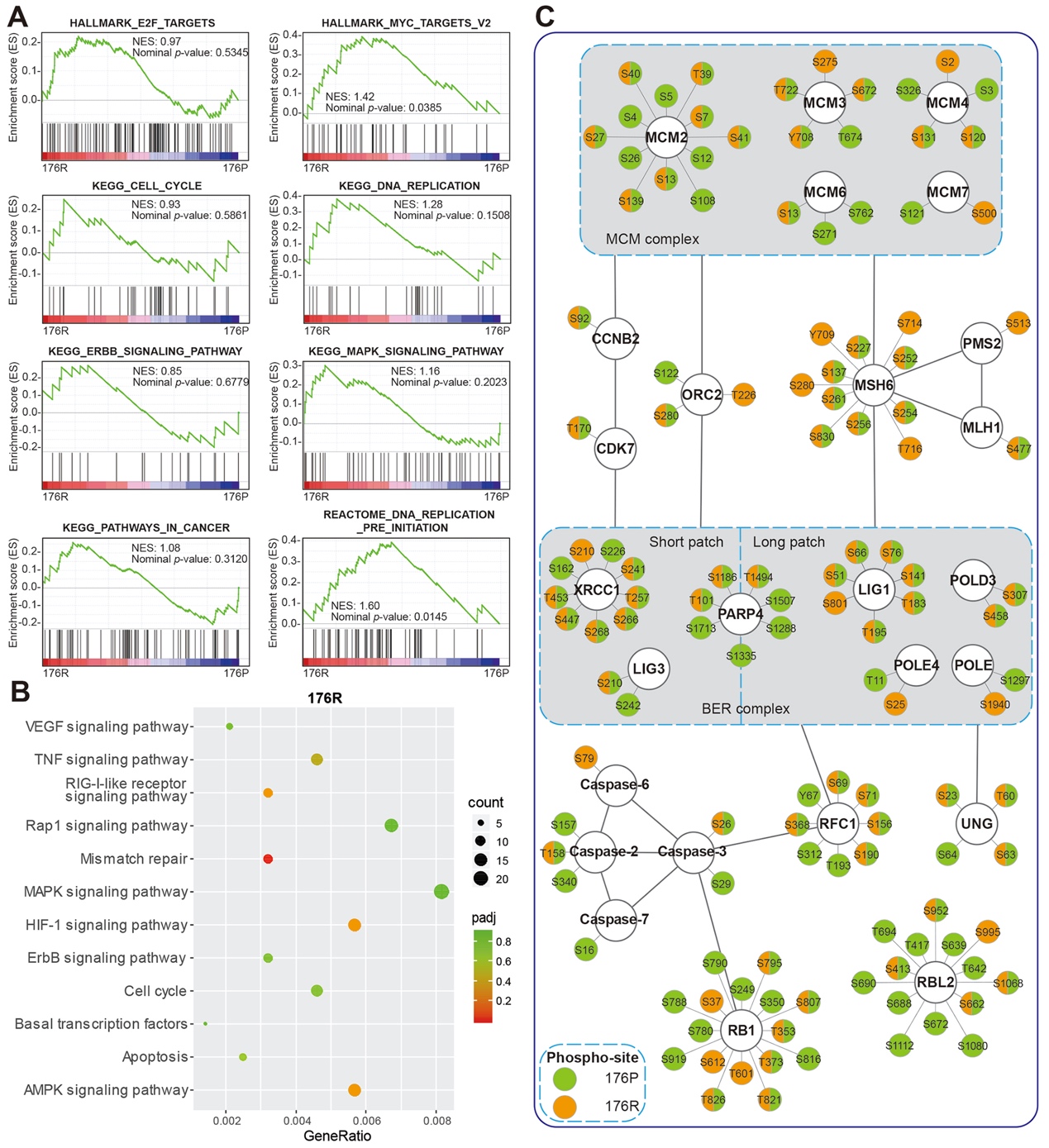
**

**Supplementary Figure S7.** **Protein expression profile and phosphorylation profile in tissues before and after trastuzumab resistance. A.** Co-expressed proteins of 176P and 176R tissues were enriched according to GO, KEGG, REACTOME, and HALLMARK datasets, by GSEA. NES: Normalized Enrichment Score. **B.** KEGG pathway enrichment of molecules that upregulated after trastuzumab resistance by protein profiling. **C.** Phosphorylation profiles of some key molecules that were involved in cell cycle, DNA replication, mismatch repair cascades.


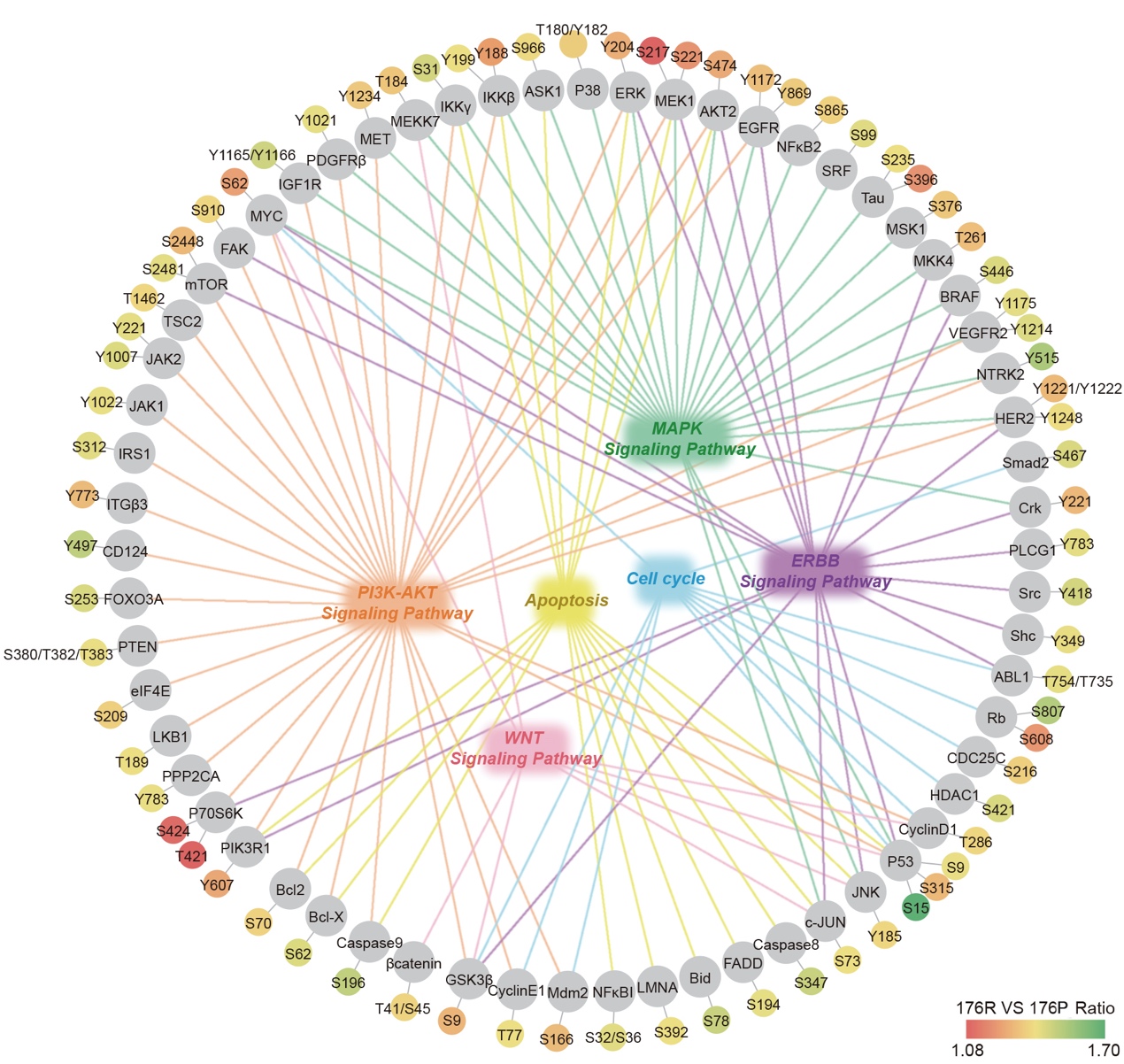


**Supplementary Figure S8. The upregulated proteins of phosphorylated forms found by protein chip.** Several critical pathways (including MAPK, PI3K/AKT, WNT pathways, cell cycle) were involved.


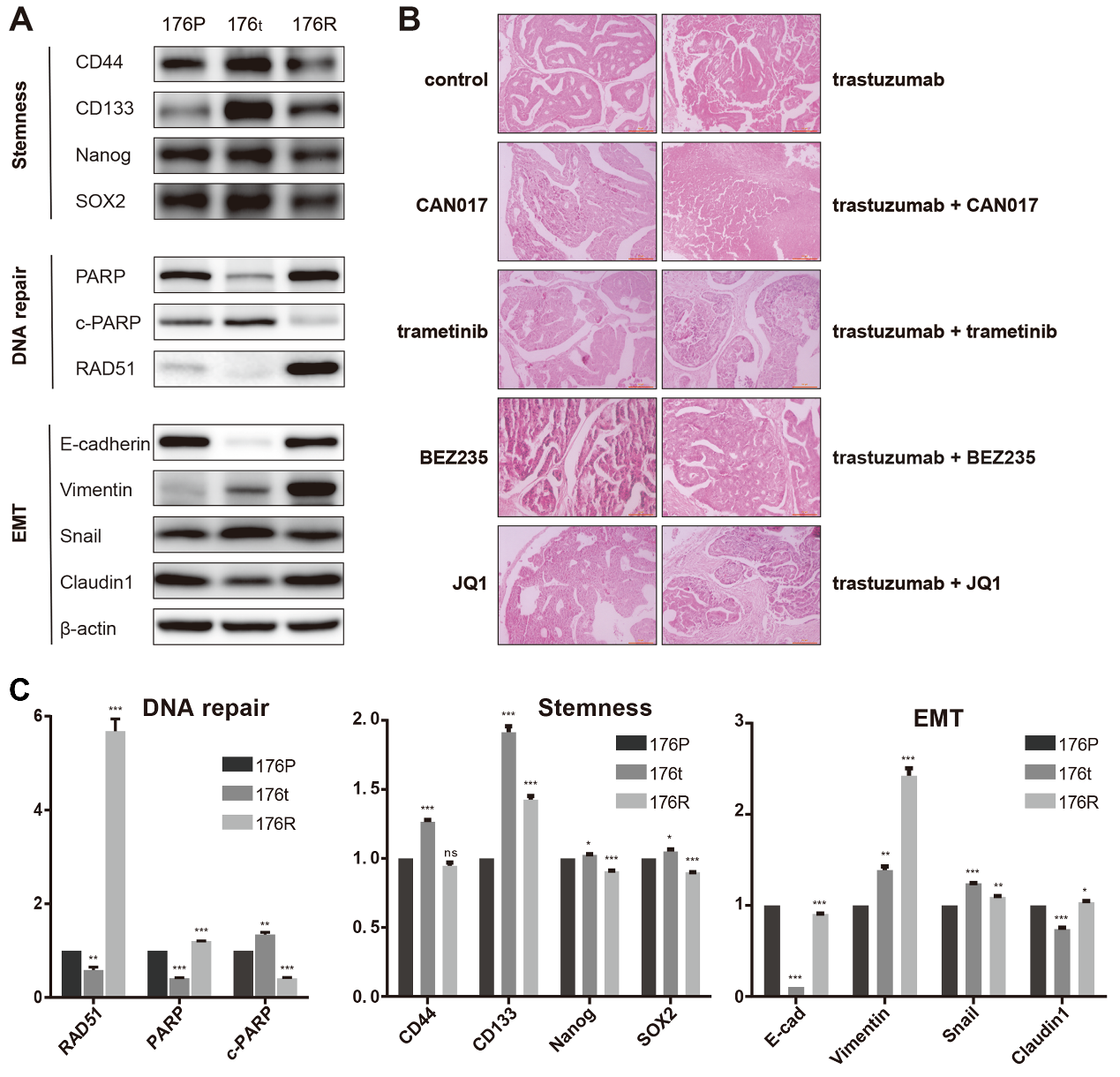


**Supplementary Figure S9. Validations of several molecules before and after trastuzumab resistance. A.** The expressions of several markers related with stemness, DNA repair, and EMT were detected by western blot. β-actin as endogenous control. **B.** HE staining (x40) of tissues in different groups, suggesting the different degrees of tumor inhibition. **C.** Quantification of WB-identified intensity of molecules related with stemness, DNA repair, and EMT.


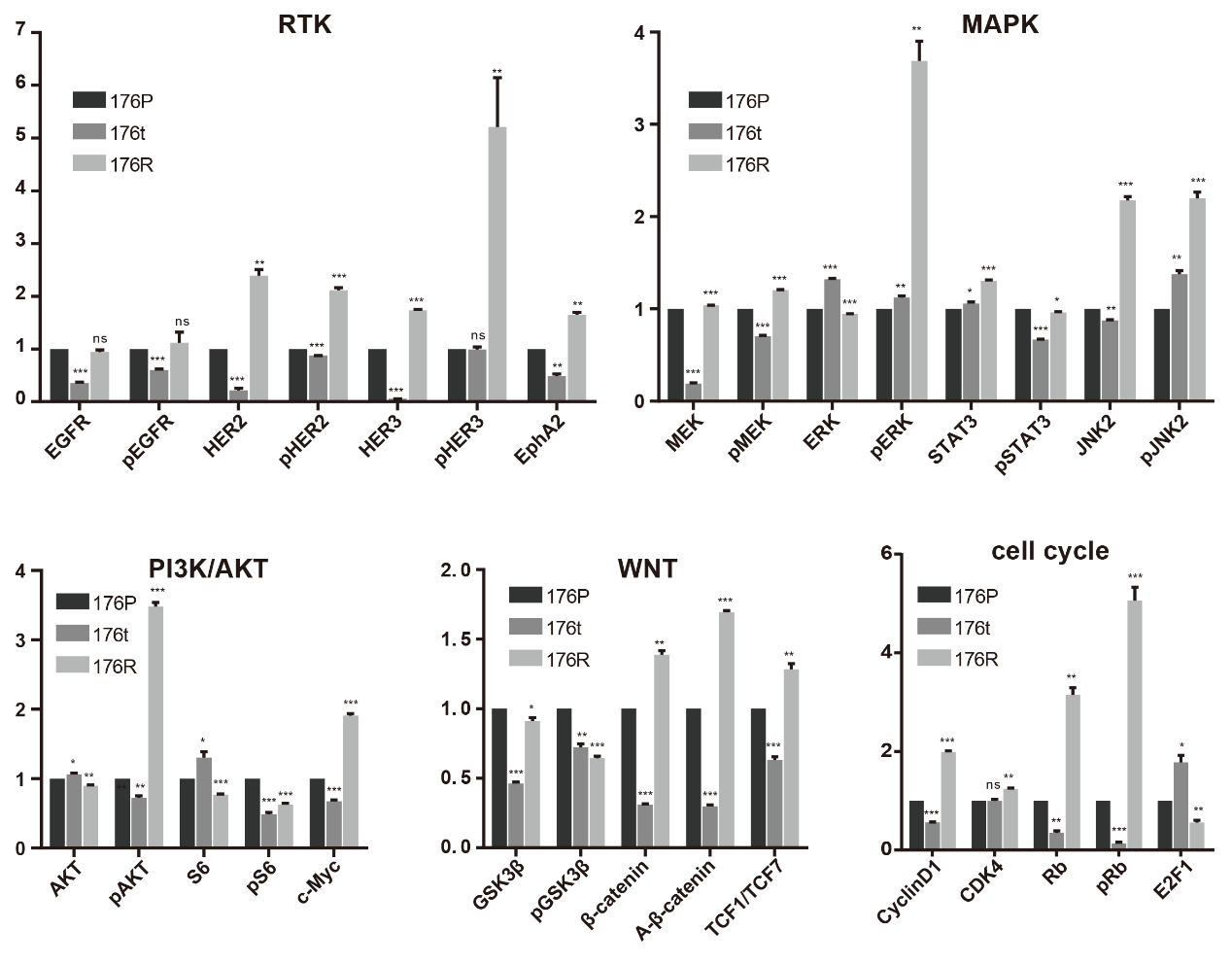


**Supplementary Figure S10. Quantification of WB-identified molecules and signaling pathways involved in Trastuzumab resistance.**


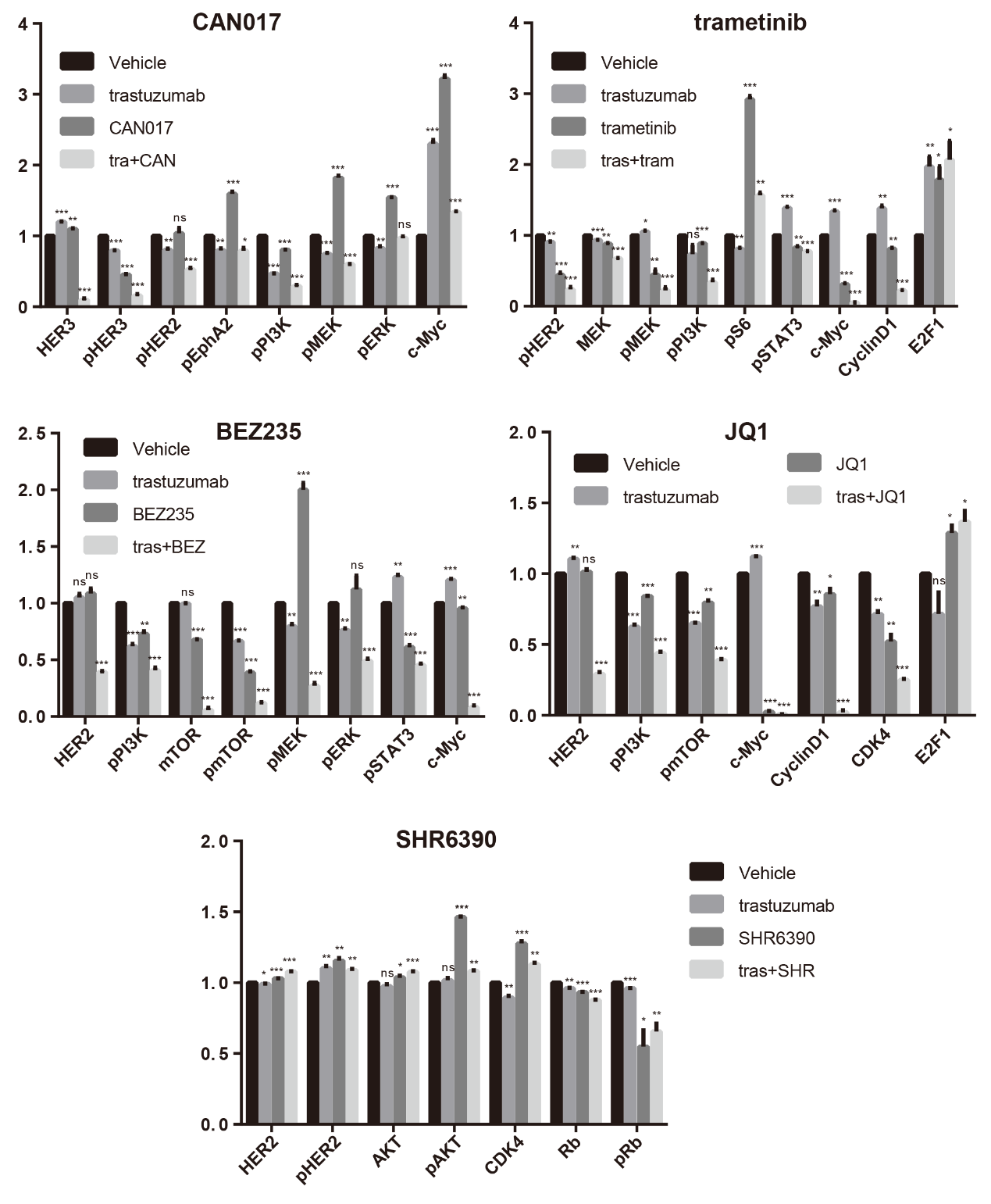


**Supplementary Figure S11. Quantification of WB-identified molecules and signaling pathways after combining inhibitors with Trastuzumab in 176R model.**

**Supplemantary Tables & Supplemantary Table Legends**

**Supplementary Table S1.** DNA targeted capture sequencing panel of 483 genes

| DNA targeted capture sequencing panel of 483 genes | | | | |
| --- | --- | --- | --- | --- |
| ABCB1 | CIC | FUBP1 | MSH6 | SF3B1 |
| ABCC1 | CSNK1A1 | FYN | MTDH | SGK1 |
| ABCC2 | COMT | FZD7 | MTHFR | SHH |
| ABCC4 | CREBBP | GALNT14 | MTOR | SIK1 |
| ABCC6 | CRKL | GATA1 | MTRR | SKP2 |
| ABCG2 | CRLF2 | GATA2 | MUTYH | SLC10A2 |
| ABL1 | CSF1R | GATA3 | MYC | SLC15A2 |
| ACK1/TNK2 | CSK | GCK | MYCL1 | SLC22A1 |
| ACVR1B | CTCF | GID4 | MYCN | SLC22A16 |
| AKT1 | CTLA4 | GINS2 | MYD88 | SLC22A2 |
| AKT2 | CTNNA1 | GNA11 | NAT1 | SLC22A6 |
| AKT3 | CTNNB1 | GNA13 | NAT2 | SLCO1B1 |
| ALK | CYBA | GNAQ | NCAM1 | SLCO1B3 |
| AMER1 | CYLD | GNAS | NCF4 | SMAD2 |
| APC | CYP19A1 | GPC3 | NCOA3 | SMAD4 |
| AR | CYP1A1 | GPR124 | NCOR1 | SMARCA4 |
| ARAF | CYP1A2 | GRIN2A | NEK11 | SMARCB1 |
| ARFRP1 | CYP1B1 | GSK3B | NF1 | SMO |
| ARID1A | CYP2A6 | GSTM1 | NF2 | SOCS1 |
| ARID1B | CYP2B6 | GSTM3 | NFE2L2 | SOD2 |
| ARID2 | CYP2C19 | GSTP1 | NFKBIA | SOX10 |
| ASXL1 | CYP2C8 | GSTT1 | NKX2-1 | SOX2 |
| ATIC | CYP2C9 | H3F3A | NOS3 | SOX9 |
| ATM | CYP2D6 | HCK | NOTCH1 | SPEN |
| ATP7A | CYP2E1 | HGF | NOTCH2 | SPG7 |
| ATR | CYP3A4 | HIF-1/HIF1A | NPM1 | SPOP |
| ATRX | CYP3A5 | HIST1H3B | NQO1 | SRC |
| AURKA | CYP4B1 | HNF1A | NRAS | SRD5A2 |
| AURKB | DAXX | HRAS | NTRK1 | SRMS |
| AXIN1 | DDR1 | HSP90AA1 | NTRK2 | STAG2 |
| AXL | DDR2 | IDH1 | NTRK3 | STAT1 |
| B2M | DNMT1 | IDH2 | NUP93 | STAT2 |
| BAIAP3 | DNMT3A | IGF1 | PAK1 | STAT3 |
| BAP1 | DOT1L | IGF1R/IGFR | PAK3 | STAT4 |
| BARD1 | DPYD | IGF2 | PALB2 | STAT5A |
| BCL2 | DSCAM | IGF2R | PARP1 | STAT5B |
| BAL2L2 | E2F1 | IKBKB | PARP2 | STAT6 |
| BCL6 | EGF | IKBKE | PAX5 | STEAP1 |
| BCOR | EGFL7 | IKZF1 | PBRM1 | STK11 |
| BCORL1 | EGFR | IL7R | PDCD1 | STK3 |
| BCR | EGR1 | INHBA | PDGFRA | STK4 |
| BIRC5 | EMC8 | INSR/IR | PDGFRB | SUFU |
| BLK | EML4 | IRF4 | PDK1 | SULT1A1 |
| BLM | ENOSF1 | IRS2 | PHF6 | SULT1A2 |
| BRAF | EP300 | ITK | PHKA2 | SULT1C4 |
| BRCA1 | EPH/EPHA1 | JAK1 | PIGF | SYK |
| BRCA2 | EPHA2 | JAK2 | PIK3CA | TCF7L1 |
| BRIP1 | EPHA3 | JAK3 | PIK3CB | TCF7L2 |
| BRK/PTK6 | EPHA4 | JUN | PIK3CG | TEK |
| BSG/CD147 | EPHA5 | KAT6A | PIK3R1 | TET2 |
| BTK | EPHA7 | KDM5A | PIK3R2 | TGFBR1 |
| C11orf30 | EPHA8 | KDM5C | PKC/PRRT2 | TGFBR2 |
| C18orf56 | EPHB1 | KDM6A | PKCγ/PRKCG | TK1 |
| C8orf34 | EPHB2 | KDR/VEGFR | PKCε/PRKCE | TMPRSS2 |
| CAMK2G | EPHB3 | KEAP1 | PLK1 | TNF |
| CAMKK2 | EPHX1 | KIT | PPARD | TNFAIP3 |
| CARD11 | ERBB2/HER2 | KLC3 | PPP1R13L | TNFRSF14 |
| CASP8 | ERBB3 | KLHL6 | PPP2R1A | TNFRSF8 |
| CBFB | ERBB4 | KMT2A/MLL | PRDM1 | TNFSF11 |
| CBL | ERCC1 | KMT2B/MLL4 | PRDX4 | TNFSF13B |
| CBR1 | ERCC2 | KMT2C/MLL3 | PRKAA1 | TOP1 |
| CBR3 | ERG | KMT2D/MLL2 | PRKAR1A | TP53 |
| CCND1 | ESR1/ER | KRAS | PRKCA | TPMT |
| CCND2 | ETV1 | LCK | PRKCB | TPX2 |
| CCND3 | ETV4 | LIMK1 | PRKDC | TRAIL-R1/TNFRSF10A |
| CCNE1 | ETV5 | LMO1 | PTCH1 | TRAIL-R2/TNFRSF10B |
| CCR4 | ETV6 | LRP1B | PTEN | TSC1 |
| CD19 | EWSR1 | LRP2 | PTK2 | TSC2 |
| CD22 | EZH2 | LYN | PTPN11 | TSHR |
| CD274 | FAM46C | MAP2K1 | PTPRD | TYMS/TS |
| CD33 | FANCA | MAP2K2 | RAC2 | TYRO3 |
| CD38 | FANCC | MAP2K4 | RAD50 | U2AF1 |
| CD3EAP | FANCD2 | MAP3K1 | RAD51 | UBE2I |
| CD52 | FANCE | MAP4K4 | RAF1 | UGT1A1 |
| CD74 | FANCF | MAP4K5 | RARA | UGT1A9 |
| CD19A | FANCG | MAPK1 | RB1 | UGT2B15 |
| CD79B | FANCL | MAPK10 | RET | UGT2B17 |
| CDA | FBXW7 | MAPK14 | RICTOR | UGT2B7 |
| CDC73 | FCGR3A | MAPK8 | RMDN2 | UMPS |
| CDH1 | FGF10 | MAPK9 | RNF43 | VEGFA |
| CDK1 | FGF14 | MAPKAPK2 | ROCK1 | VEGFB |
| CDK12 | FGF19 | MARK1 | RON/MST1R | VHL |
| CDK2 | FGF23 | MCL1 | ROS1 | WEE1 |
| CDK4 | FGF3 | MDM2 | RPL13 | WISP3 |
| CDK5 | FGF4 | MDM4 | PRS6KA1 | WNK3 |
| CDK6 | FGF6 | MED12 | RPS6KB1 | WT1 |
| CDK7 | FGFR1 | MEF2B | RPTOR | XPC |
| CDK8 | FGFR2 | MEN1 | RRM1 | XPO1 |
| CDK9 | FGFR3 | MERTK | RUNX1 | XRCC1 |
| CDKN1B | FGFR4 | MET | SCF/KITLG | XRCC4 |
| CDKN2A | FGR | MITF | SDHA | YES1 |
| CDKN2B | FKBP1A | MKNK2 | SDHAF1 | ZAP70 |
| CDKN2C | FLT1 | MLH1 | SDHAF2 | ZC3HAV1 |
| CEBPA | FLT3 | MPL | SDHB | ZNF217 |
| CHEK1 | FLT4 | MRE11A | SDHC | ZNF703 |
| CHEK2 | FOXL2 | MS4A1 | SDHD |  |
| CHST3 | FRK | MSH2 | SETD2 |  |

**Supplementary Table S2.** Antibodies involved in western blot and immunohistochemistry

| **Antibodies** | **Catalog Number** | **Manufacturer** | **Western Blot (Dilution)** | **Immunohistochemistry (Dilution)** |
| --- | --- | --- | --- | --- |
| HER2 | #2165 | Cell Signaling Technology | 1:1000 | 1:400 |
| pHER2 (Tyr1221/1222) | #2243 | Cell Signaling Technology | 1:1000 |  |
| AKT | #4691 | Cell Signaling Technology | 1:1000 |  |
| pAKT (Ser473) | #4060 | Cell Signaling Technology | 1:2000 |  |
| S6 | #2217 | Cell Signaling Technology | 1:1000 |  |
| pS6 (Ser235/236) | #4858 | Cell Signaling Technology | 1:2000 |  |
| EGFR | #4267 | Cell Signaling Technology | 1:1000 |  |
| pEGFR (Tyr1068) | #3777 | Cell Signaling Technology | 1:1000 |  |
| HER3 | #12708 | Cell Signaling Technology | 1:1000 |  |
| pHER3 (Tyr1289) | #2842 | Cell Signaling Technology | 1:1000 |  |
| mTOR | #2983 | Cell Signaling Technology | 1:1000 |  |
| pmTOR (Ser2448) | #5536 | Cell Signaling Technology | 1:1000 |  |
| pPI3K p85 (Tyr458) / p55 (Tyr199) | #17366 | Cell Signaling Technology | 1:1000 |  |
| EphA2 | #6997 | Cell Signaling Technology | 1:1000 |  |
| pEphA2 (Tyr588) | #6347 | Cell Signaling Technology | 1:1000 |  |
| MEK1/2 | #9126 | Cell Signaling Technology | 1:1000 |  |
| pMEK1/2 (Ser217/221) | #9154 | Cell Signaling Technology | 1:1000 |  |
| ERK1/2 | #4695 | Cell Signaling Technology | 1:1000 |  |
| pERK1/2 (Thr202/Tyr204) | #4370 | Cell Signaling Technology | 1:2000 |  |
| JNK2 | #9258 | Cell Signaling Technology | 1:1000 |  |
| pJNK2 (Thr183/Tyr185) | ab4821 | Abcam | 1:1000 |  |
| c-Myc | ab32072 | Abcam | 1:1000 | 1:100 |
| TCF1/TCF7 | #2203 | Cell Signaling Technology | 1:1000 | 1:400 |
| STAT3 | #9139 | Cell Signaling Technology | 1:1000 |  |
| pSTAT3 (Tyr705) | #9145 | Cell Signaling Technology | 1:2000 |  |
| E2F1 | ab179445 | Abcam | 1:1000 |  |
| CyclinD1 | #2978 | Cell Signaling Technology | 1:1000 |  |
| CDK4 | #12790 | Cell Signaling Technology | 1:1000 |  |
| CDK6 | #13331 | Cell Signaling Technology | 1:1000 |  |
| Rb | #9309 | Cell Signaling Technology | 1:2000 |  |
| pRb (Ser807/811) | #8516 | Cell Signaling Technology | 1:1000 |  |
| β-catenin | #8480 | Cell Signaling Technology | 1:1000 |  |
| A-β-catenin | #19807 | Cell Signaling Technology | 1:1000 |  |
| p-β-catenin (Ser33/37/Thr41) | #9561 | Cell Signaling Technology | 1:1000 |  |
| GSK3β | #12456 | Cell Signaling Technology | 1:1000 |  |
| pGSK3β (Ser9) | #5558 | Cell Signaling Technology | 1:1000 |  |
| β-actin | #014M4759 | Sigma | 1:10000 |  |
| Anti-Rabbit IgG, HRP-linked antibody | #7074 | Cell Signaling Technology | 1:1000 |  |
| Anti-Mouse IgG, HRP-linked antibody | #7076 | Cell Signaling Technology | 1:1000 |  |
| Enzyme-labeled goat anti-mouse/rabbit IgG polymer | #PV-6000 | ZSGB-BIO |  | / |
| Ki67 | #ZM-0167 | ZSGB-BIO |  | 1:50 |

**Supplementary Table S3.** The weight change of mice under different treatments

| **Experiment 1** | | | | | | | | | | | | | | | | |
| --- | --- | --- | --- | --- | --- | --- | --- | --- | --- | --- | --- | --- | --- | --- | --- | --- |
| **Treatment** | **Day0** | | **Day3** | | **Day6** | | **Day9** | | **Day12** | | **Day15** | | **Day18** | | **Day21** | |
|  | **mean (%)** | **SD (%)** | **mean (%)** | **SD (%)** | **mean (%)** | **SD (%)** | **mean (%)** | **SD (%)** | **mean (%)** | **SD (%)** | **mean (%)** | **SD (%)** | **mean (%)** | **SD (%)** | **mean (%)** | **SD (%)** |
| **vehicle** | 100 | 0 | 103 | 0.23 | 101.5 | 0.99 | 102.5 | 6.53 | 100.8 | 1.99 | 99.76 | 1.37 | 97.53 | 1.73 | 96.41 | 1.36 |
| **trastuzumab** | 100 | 0 | 103.5 | 2.94 | 102 | 0.94 | 100.1 | 1.06 | 97.4 | 1.81 | 95.91 | 1.53 | 96.42 | 1.94 | 95.42 | 2.13 |
| **CAN017** | 100 | 0 | 103.8 | 2.83 | 104 | 3.09 | 101.9 | 1.41 | 100.9 | 2.06 | 98.48 | 3 | 95.2 | 2.68 | 95.34 | 0.91 |
| **tras+CAN** | 100 | 0 | 102.9 | 1.36 | 104 | 1.43 | 96.61 | 3.94 | 96.61 | 4.26 | 91.56 | 4.18 | 96.34 | 1.34 | 97.39 | 1.33 |
| **trametinib** | 100 | 0 | 97.21 | 1.05 | 94.5 | 0.86 | 93.14 | 1.91 | 91.73 | 0.14 | 86.3 | 1.9 | 84.96 | 2.08 | 85.86 | 2.59 |
| **tras+tram** | 100 | 0 | 96.18 | 0.42 | 89.76 | 1.06 | 89.04 | 1.23 | 89.47 | 1.35 | 88.78 | 1 | 85.83 | 0.87 | 85.65 | 1.45 |
| **JQ1** | 100 | 0 | 95.44 | 1.52 | 86.56 | 0.78 | 83.82 | 1.82 | 80.86 | 2.73 | 83.54 | 2.95 | 81.08 | 1.88 | 83.43 | 2.09 |
| **tras+JQ1** | 100 | 0 | 94.69 | 1.82 | 88.84 | 3.97 | 91.02 | 2.39 | 90.3 | 1.75 | 85.5 | 3.11 | 82.29 | 4.24 | 82.03 | 3.46 |
| **BEZ235** | 100 | 0 | 93.36 | 0.52 | 87.86 | 1.29 | 82.26 | 3.34 | 85.8 | 3.24 | 84.08 | 2.44 | 82.47 | 4.84 | 76.88 | 0.96 |
| **tras+BEZ** | 100 | 0 | 92.57 | 1.85 | 88.52 | 1.76 | 83.86 | 2.12 | 83.44 | 2.67 | 81.97 | 1.64 | 86.02 | 0.44 | 79 | 2.28 |

| **Experiment 2** | | | | | | | | | | | | | | | | |
| --- | --- | --- | --- | --- | --- | --- | --- | --- | --- | --- | --- | --- | --- | --- | --- | --- |
| **Treatment** | **Day0** | | **Day3** | | **Day6** | | **Day9** | | **Day12** | | **Day15** | | **Day18** | | **Day21** | |
|  | **mean (%)** | **SD (%)** | **mean (%)** | **SD (%)** | **mean (%)** | **SD (%)** | **mean (%)** | **SD (%)** | **mean (%)** | **SD (%)** | **mean (%)** | **SD (%)** | **mean (%)** | **SD (%)** | **mean (%)** | **SD (%)** |
| **vehicle** | 100 | 0 | 101.8 | 1.3 | 100.8 | 1.22 | 99.56 | 1.86 | 101.7 | 1.81 | 100.2 | 1.25 | 101.3 | 1.41 | 102 | 2.07 |
| **trastuzumab** | 100 | 0 | 102.1 | 1.68 | 103.8 | 1.41 | 102.4 | 1.16 | 101.3 | 1.26 | 99.55 | 1.24 | 100.8 | 1.66 | 103.5 | 2.4 |
| **SHR6390** | 100 | 0 | 90.2 | 3.77 | 92.69 | 2.35 | 86.39 | 2.01 | 87.02 | 0.82 | 86.55 | 0.59 | 87.61 | 1.52 | 88.8 | 2.96 |
| **tras+SHR** | 100 | 0 | 97.73 | 3.87 | 100.5 | 2.95 | 94.51 | 1.31 | 95.02 | 1.21 | 93.61 | 2.99 | 91.8 | 3.08 | 89.43 | 3.41 |
